# Erythropoietin Activates Autophagy to Regulate Apoptosis and Angiogenesis of Periodontal Ligament Stem Cells via the Akt/ERK1/2/BAD Signaling Pathway under Inflammatory Microenvironment

**DOI:** 10.1155/2022/9806887

**Published:** 2022-09-20

**Authors:** Denghao Huang, Jie Lei, Xingrui Li, Zhonghao Jiang, Maoxuan Luo, Yao Xiao

**Affiliations:** ^1^Luzhou Key Laboratory of Orofacial Reconstruction and Regeneration, The Affiliated Stomatology Hospital of Southwest Medical University & Institute of Stomatology, Southwest Medical University, Luzhou, China; ^2^Cell Biology Technology Platform, Public Experiment Technology Center of Southwest Medical University, Luzhou, China; ^3^Department of Chengbei Outpatient, The Affiliated Stomatology Hospital of Southwest Medical University, Luzhou, China; ^4^Department of Orthodontics, The Affiliated Stomatology Hospital of Southwest Medical University, Luzhou, China

## Abstract

**Background:**

Angiogenic tissue engineering is a vital problem waiting to be settled for periodontal regeneration. Erythropoietin, a multieffect cytokine, has been reported as a protective factor for cell fate. According to our previous study, erythropoietin has a significantly angiogenic effect on periodontal ligament stem cells. To further explore its potential effects and mechanism, we studied biological behaviors of periodontal ligament stem cells under inflammatory microenvironment induced by different concentrations (0, 10, 20, 50, and 100 ng/mL) of tumor necrosis factor-*α* (TNF-*α*) and examined how different concentrations (0, 5, 10, 20, and 50 IU/mL) of erythropoietin changed biological behaviors of periodontal ligament stem cells.

**Materials and Methods:**

Cell Counting Kit-8 was used for cell proliferation assay. Annexin V-PI-FITC was used for cell apoptosis through flow cytometry. Matrigel plug was adopted to measure the angiogenic capacity *in vitro*. RNA sequencing was used to detect the downstream signaling pathway. Quantitative real-time polymerase chain reaction was conducted to examine mRNA expression level. Western blot and immunofluorescence were applied to testify the protein expression level.

**Results:**

Periodontal ligament stem cells upregulated apoptosis and suppressed autophagy and angiogenesis under inflammatory microenvironment. Erythropoietin could activate autophagy to rescue apoptosis and angiogenesis levels of periodontal ligament stem cells through the Akt/Erk1/2/BAD signaling pathway under inflammatory microenvironment.

**Conclusions:**

Erythropoietin could protect periodontal ligament stem cells from inflammatory microenvironment, which provided a novel theory for periodontal regeneration.

## 1. Introduction

Periodontitis, a commonly infectious oral disease, is deemed as the sixth disease of the globe, which would risk psychological and physical health [1]. Periodontitis is characterized as loss of periodontal tissues and detachment of teeth, which is caused by a series of microbiological and immune factors [2, 3]. Hitherto, there is still lack of effective therapy to reverse the periodontal loss [4, 5]. Many researchers have turned to odontogenic stem cells for novel strategies [6], such as periodontal ligament stem cells (PDLSCs) [7] and apical papilla stem cells (APSCs) [8]. PDLSCs have shown the great ability of multidirectional differentiation, especially in osteogenesis and angiogenesis [9]. However, two crucial problems are waiting to be solved. Firstly, it is essential to reverse the differentiational capacity of PDLSCs, which is always undermined by inflammatory environment. Secondly, it is vital to regenerate vascularized bone in periodontal regeneration, which offers nutrition to the bone [10].

Tissue reconstruction also relies on the cytokines. Erythropoietin (EPO), a glycoprotein secreted by the kidney, is reported as a promising candidate cytokine for periodontal tissue engineering. EPO plays a great effect on osteogenic and angiogenic differentiation of mesenchymal stem cells (MSCs) [11, 12]. It is widely investigated that biomaterials loaded with EPO could promote osteogenesis and angiogenesis of bone marrow stem cells (BMSCs) and vein endothelial cells (VECs) through upregulating the EphB4/EphrinB2 signaling pathway [13–15]. Furthermore, EPO regulates Wnt/*β*-catenin and p38/MAPK signaling pathway to enhance osteogenesis of PDLSCs [16, 17]. EPO is also seen as an anti-inflammation factor, which provides a stable microenvironment for tissue engineering. Relative researches have proven that oxidative stress and cell apoptosis can be improved by EPO through reducing IL1*β*, iNOS, and CD68 expression [18, 19]. Collectively, EPO dually regulates multidirectional differentiation and anti-inflammation effects, which could be used for periodontal regeneration.

Cumulative studies have proved that autophagy is closely related to cell differentiation and apoptosis. Autophagy is considered as a helpful process for cell viability, which could enable cells to adapt to the change and pressure of circumstance [20]. Jiang et.al [21] explored the relationship between autophagy and Akt signaling pathway, which indicated that increasing LC3 expression could enhance the remodeling of alveolar bone. Autophagy decreased apoptosis and retained the osteogenic ability of PDLSCs and osteoblasts [22, 23]. Angiogenesis of PDLSCs could also be induced *via* upregulating autophagy [24]. Researchers also found that higher autophagy-related gene expression of LC3, Beclin1, Atg7, and Atg12 protected PDLSCs from apoptosis in inflammatory microenvironment [25]. It is evidenced that autophagy could be activated through overexpression of the tumor necrosis factor alpha-induced protein 3 (TNFAIP3) to diminish inflammation of periodontal ligament cells (PDLCs) induced by lipopolysaccharide (LPS) and nicotine [26].

The Akt signaling pathway always participates in autophagy, apoptosis, and differentiation. Akt-related autophagy maintains the stemness of mouse embryonic palatal mesenchymal stem cells, which could suppress cleft palate development [27]. It has been discussed that autophagy could be augmented by upregulating the Akt signaling pathway to regulate cell fate [28–30]. The Akt signaling pathway is also involved in the angiogenesis of BMSCs, VECs, and adipose-derived stem cells (ASCs) to promote fracture repair [31–34].

According to the existing literatures and our previous study, we aimed to investigate the antiapoptosis and angiogenesis of EPO under inflammatory microenvironment. We also explored the involvement of autophagy and Akt signaling pathway in this process, wishing to find a novel approach for periodontal repair.

## 2. Methods and Materials

### 2.1. Isolation and Cultivation of PDLSCs

With fully informed consent, we collected 50 healthy orthodontic teeth from adolescent patients (12-20 years old) under the approval of the Biomedical Ethics Committee of the Affiliated Stomatology Hospital of Southwest Medical University (Lot No. 2020112600). After collecting teeth, the periodontal ligaments were scraped from the tooth root and incubated reversely for 4 hours in a culture flask. The medium, mixture of *α*-MEM (Gibco, CA, USA), 10% fetal bovine serum (FBS) (EveryGreen, Shanghai, China), and 1% penicillin-streptomycin solution (Beyotime, Shanghai, China), was added into the flask to obtain periodontal ligament stem cells. The cells usually crawled out about 2 weeks later. The culturing incubator (Thermo Fisher, CA, USA) was set as 37°C and 5% CO_2_.

### 2.2. Flow Cytometry Detection for Surface Marker

After digesting and washing, the fourth-generation (p4) PDLSCs were used to detect specific surface markers under flow cytometry instrument (BD Biosciences, NJ, USA). Mesenchymal stem cell surface antibodies (CD90, CD44, and CD105) (BD Biosciences, NJ, USA) and hematopoietic stem cell surface antibodies (CD45, CD31) (BD Biosciences, NJ, USA) were selected to examine.

### 2.3. Osteogenic and Lipogenic Induction Assays

Osteogenesis and lipogenesis induction were used to detect the multidirectional differentiation ability of PDLSCs. The osteogenic induction solution formulation consisted of D-MEM (Hyclone, USA), 10% FBS, 10 mmol/L sodium *β*-glycerate (Macklin, Shanghai, China), 0.1 *μ*mol/L dexamethasone (Solarbio, Beijing, China), and 50 mg/L vitamin C (Solarbio, Beijing, China). Osteogenic induction solution was changed every 3 days and maintained for 28 days. Then, cells were fixed by paraformaldehyde and stained by alizarin red solution (Solarbio, Beijing, China). The lipogenic induction solution was formulated with D-MEM, 10% FBS, 10 *μ*mol/L dexamethasone (Solarbio, Beijing, China), 200 *μ*mol/L indomethacin (Solarbio, Beijing, China), 0.5 mmol/L 3-isobutyl-1-methylxanthine (IBMX) (Sigma, USA), and 10 mg/L insulin (Solarbio, Beijing, China). Cultured for 28 days, the cells were fixed and stained by oil red O solution (Solarbio, Beijing, China). Calcium nodules and lipid droplets were observed under a fluorescent inverted microscope (Olympus, Japan).

### 2.4. Cell Proliferation Assay

Cell Counting Kit-8 (CCK8) (Dojindo, Japan) was used for cell proliferation assay. The p4 PDLSC was inoculated in 96-well plates at a density of 2000 cells per well, and the cells were divided into different treated groups. Detection was performed on days 1, 3, 5, and 7 after inoculation, respectively. Overall, 10 *μ*L of CCK8 solution and 90 *μ*L *α*-MEM were added to each well, incubated for one hour and then detected at 450 nm absorbance in an enzyme microplate reader (BioTek, USA).

### 2.5. Annexin V-FITC-PI Double-Staining Assay (Cell Apoptosis Assay)

Annexin V-FITC-PI double-staining assay was performed under flow cytometry to detect cell apoptosis level. Cells were collected after incubating for 1 day and diffused in 400 *μ*L binding buffer. Then, PDLSCs were stained with 2 *μ*L Annexin V and 1 *μ*L propidium iodide (PI) (BD Biosciences, NJ, USA) and incubated for 30 min.

### 2.6. Total RNA Extraction and Real-Time Quantitative Polymerase Chain Reaction (qPCR)

Total RNA was extracted according to the instructions of the Total RNA Extraction Kit (TIANGEN, Beijing, China), and then, RNA was reverse transcribed to cDNA using the Takara Reverse Transcription Kit (TOYOBO, Tokyo, Japan) for subsequent experiment. We prepared a 20 *μ*L amplification system using the SYBR FAST qPCR Master Mix Kit (TOYOBO, Tokyo, Japan) and then performed amplification in Bio-Rad/CFX96 fluorescence quantitative PCR instrument (Bio-Rad, USA). The specific conditions of denaturation, annealing, and extension were as follows: 95°C for 3 min, 95°C for 5 sec, 56°C for 10 sec, and 72°C for 25 sec in 40 cycles. The forward and reverse primer (BI, Shanghai, China) sequences used in the experiment are shown in Table 1. The relative expression of target genes was normalized to the expression of *β*-actin, and the changes in gene expression were calculated by the 2-^△△^CT method.

### 2.7. Total Protein Extraction and Western Blot Assay

Total protein was extracted under the guidance of the kit (Solarbio, Beijing, China) instructions. The protein concentration of each group was determined using the BCA protein concentration assay kit (Solarbio, Beijing, China). SDS-PAGE gels were configured, and the proteins were electrophoresed vertically and transferred to PVDF membranes (Millipore, Germany). The membranes were blocked for 2 hours in blocking buffer (Solarbio, Beijing, China) at room temperature. Then, membranes were incubated with primary antibody at 4°C overnight. Washed in TBST solution for 3 times, membranes were incubated with anti-rabbit IgG, HRP-linked antibody (#7074, CST, USA) at room temperature for 1 hour. ECL developer (Absin, Shanghai, China) was added and photographed in a chemiluminescence imaging system (Tanon, Shanghai, China). The primary antibodies are listed in Table 2 (Supplementary Figure 1, raw data of western blot).

### 2.8. Matrigel Plug Assay

Melted matrix gel (Corning, USA) was evenly added in the volume of 50 *μ*L to the precooled 96-well plate and placed in the incubator for 30 minutes. Pretreated PDLSCs were added to the wells at a density of 2000 cells per well. Tube formation in each well was observed after 6 hours and photographed under fluorescent inverted microscope.

### 2.9. RNA-Sequencing Assay

The transcriptome expression of PDLSCs in control groups (TNF-*α* treatment) and treated groups (TNF-*α* and EPO treatment) was examined through mRNA sequencing to discover downstream regulatory pathways of EPO. Each group had 3 biological replicates. RNA extraction, specific RNA library preparation, RNA sequencing, and bioinformatics analysis were done by OE biotech Co., Ltd. (Shanghai, China). The sequence raw data has been submitted to NCBI Sequence Read Archive (Accession number PRJNA824457). The sequence results have been validated by qPCR.

### 2.10. Immunofluorescence and Confocal Laser Microscope Observation

PDLSCs were treated under different conditions on the cell climbing sheets for 1 day. Fixed by paraformaldehyde, 1000 *μ*L blocking buffer (0.2% Triton-100 and 5% donkey serum) was added onto each sheet. Then, the cells were incubated with the primary antibody (VEGF-a or LC3B) at 4°C overnight. After rewarming to room temperature, the cells were dealt with the secondary antibody, DyLight680 (Invitrogen, CA, USA) for 1 hour. DAPI and phalloidin (CoraLite488, Proteintech, Wuhan, China) were used to stain cell nucleus and cytoskeleton. Images of cells were observed and captured under Olympus SpinSR confocal laser microscope (Olympus, Tokyo, Japan).

### 2.11. Study Design

All experiments were all performed under p4 PDLSCs. Experiment groups of this research were mainly divided into 4 parts:

1. Concentration gradient of TNF-*α* (treated for 3 days): 0, 10, 20, 50, and 100 ng/mL
2. Concentration gradient of EPO (under inflammatory microenvironment induced by 20 ng/mL TNF-*α*) (treated for 3 days): 0, 5, 10, 20, and 50 IU/mL
3. To explore the roles of the Akt/ERK1/2/BAD signaling pathway (treated for 1 day): ① TNF-*α* (50 ng/mL), ② TNF-*α* (50 ng/mL)+LY294002 (10 *μ*M)+EPO (20 IU/mL), and ③ TNF-*α* (50 ng/mL)+EPO (20 IU/mL)
4. To explore the roles of autophagy (treated for 1 day): ① TNF-*α* (50 ng/mL), ② TNF-*α* (50 ng/mL)+3-methyladenine (3-MA) (5 mM)+EPO (20 IU/mL), and ③ TNF-*α* (50 ng/mL)+EPO (20 IU/mL)

### 2.12. Statistical Analysis

Statistical calculation was completed at GraphPad Prism 9.0 software (GraphPad, CA, USA). Results were presented in the form of mean ± standard deviation (SD). Each experiment has been performed at least three times. One-way ANOVA was used to determine multiple-group comparisons. And Students' *t*-test was used to compare among two groups. It was considered as statistically significant when *P* < 0.05.

## 3. Results

### 3.1. Identification of PDLSCs and Establishment of Inflammatory Microenvironment

**Figure 1:**
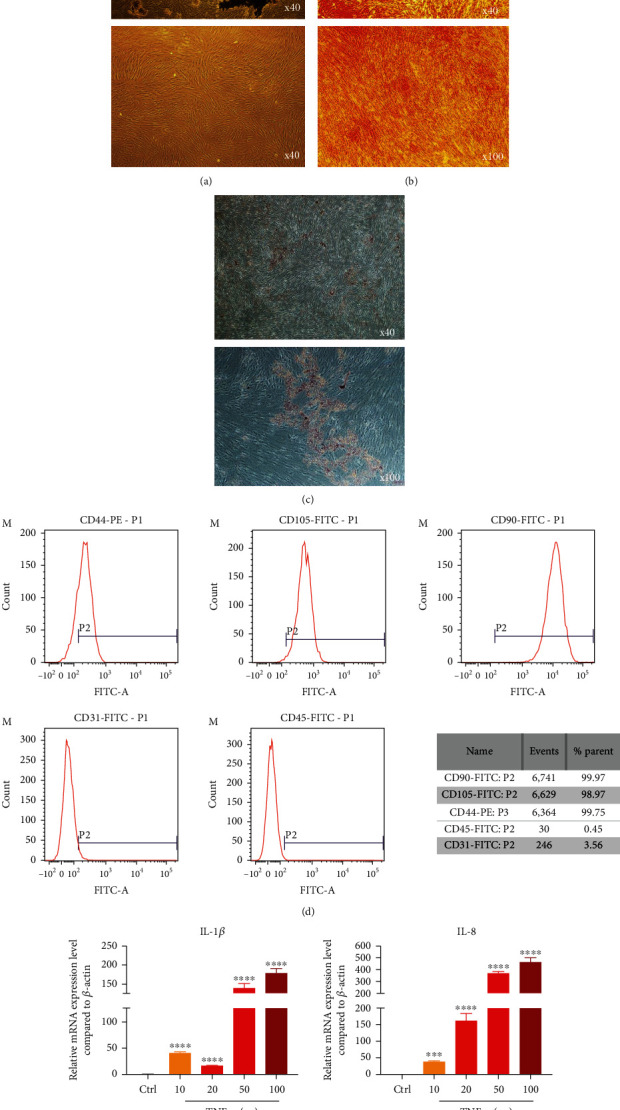
Identification of PDLSCs and establishment of inflammatory microenvironment. (a) Primary passage PDLSCs derived from periodontal ligament and the first passage PDLSCs digested from primary passage. (b) Observed osteogenic induction of PDLSCs under fluorescent inverted microscope. (c) Observed lipogenic induction of PDLSCs under fluorescent inverted microscope. (d) Flow cytometry detected cell surface marker (CD44, CD105, CD90, CD31, and CD45). (e) mRNA expression level of IL-1*β* and IL-8 compared to *β*-actin through qPCR. Data are presented as mean ± SD (*n* = 3); ^∗^*P* < 0.05, ^∗∗^*P* < 0.01, ^∗∗∗^*P* < 0.001, and ^∗∗∗∗^*P* < 0.0001.

Cells were obtained from the periodontal ligament tissues (Figure 1(a)). Cells could differentiate into osteogenesis and adipogenesis under induction, which suggested the capacity of multidirectional differentiation (Figures 1(b) and 1(c)). Regarding the cell surface markers, cells highly expressed specific markers of MSCs (CD90, CD44, and CD105) but rarely expressed specific markers of HSCs (CD45, CD31) (Figure 1(d)). The mRNA expression level of the inflammatory cytokines (IL-1*β*, IL-8) significantly upregulated with the ascent of TNF-*α* concentration (Figure 1(e)).

### 3.2. PDLSCs Reduced Proliferation and Upregulated Apoptosis under Inflammatory Microenvironment

**Figure 2:**
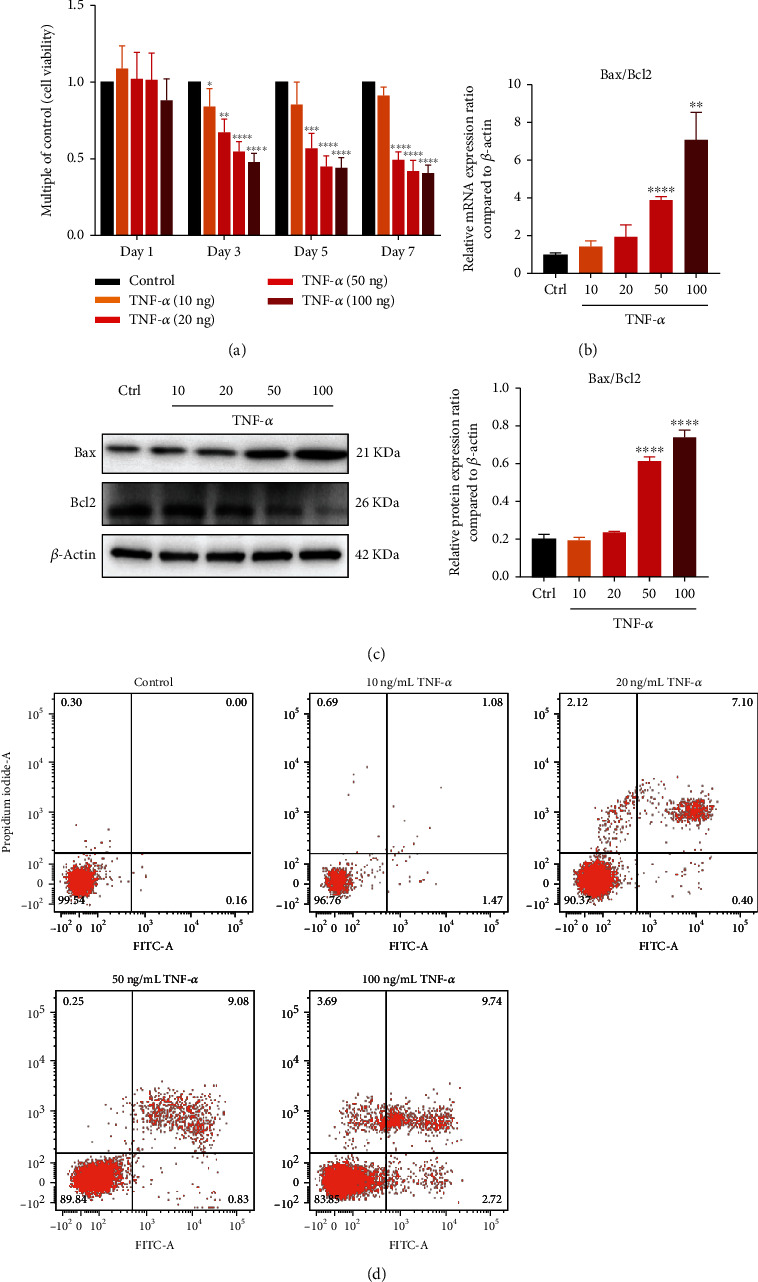
PDLSCs reduced proliferation and upregulated apoptosis under inflammatory microenvironment. (a) Cell viability of TNF-*α* treatment groups. (b) mRNA expression levels of Bax/Bcl2 ratio compared to *β*-actin through qPCR. (c) Protein expression levels of Bax/Bcl2 ratio compared to *β*-actin through western blot. (d) Cell apoptosis rate of TNF-*α* treatment groups. Data are presented as mean ± SD (*n* = 3); ^∗^*P* < 0.05, ^∗∗^*P* < 0.01, ^∗∗∗^*P* < 0.001, and ^∗∗∗∗^*P* < 0.0001.

CCK8 results showed that PDLSCs gradually reduced proliferation under different concentrations of TNF-*α* on days 1, 3, 5, and 7 (Figure 2(a)). The mRNA expression of Bax/Bcl2 ratio was significantly upregulated with the raising of TNF-*α* concentration (Figure 2(b)). The protein expression trend was the same (Figure 2(c)). Annexin V-FITC-PI assay showed the enhancement of apoptosis rate, accompanying with the increasing TNF-*α* concentration (Figure 2(d)). In a short, TNF-*α* impaired cell viability and upregulated apoptosis of PDLSCs.

### 3.3. PDLSCs Repressed Autophagy and Angiogenic Capacity under Inflammatory Microenvironment

**Figure 3:**
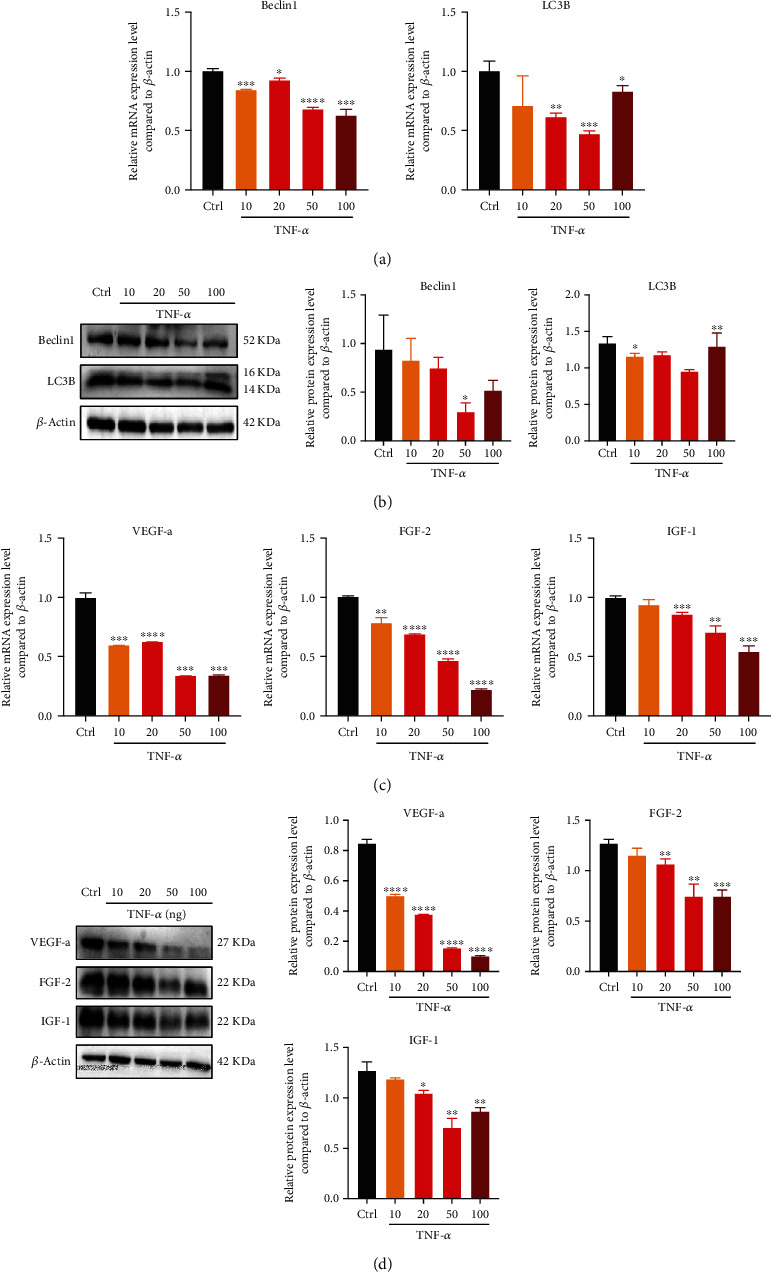
PDLSCs repressed autophagy and angiogenic capacity under inflammatory microenvironment. (a, b) mRNA and protein expression levels of Beclin1 and LC3B compared to *β*-actin through qPCR and western blot. (c, d) mRNA and protein expression levels of VEGF-a, FGF-2, and IGF-1 compared to *β*-actin through qPCR and western blot. Data are presented as mean ± SD (*n* = 3); ^∗^*P* < 0.05, ^∗∗^*P* < 0.01, ^∗∗∗^*P* < 0.001, and ^∗∗∗∗^*P* < 0.0001.

The mRNA and protein expression levels of autophagy-related cytokines (Beclin1, LC3B) indicated that TNF-*α* can significantly repress autophagy, especially in 20 and 50 ng/mL groups (Figures 3(a) and 3(b)). Additionally, the mRNA and protein expression levels of vascularization-related cytokines (VEGF-a, IGF-1, and FGF-2) descended when TNF-*α* concentration was enhanced, particularly in 20, 50, and 100 ng/mL groups (Figures 3(c) and 3(d)).

### 3.4. EPO Rescued Inflammation and Apoptosis Levels of PDLSCs under Inflammatory Microenvironment

**Figure 4:**
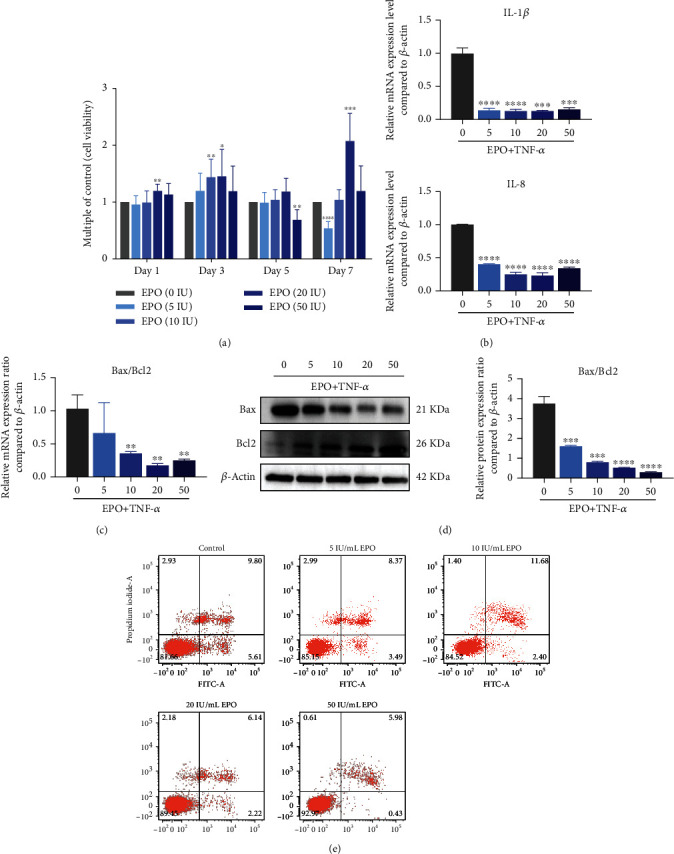
EPO rescued inflammation and apoptosis levels of PDLSCs under inflammatory microenvironment. (a) Cell viability of EPO treatment groups. (b) mRNA expression levels of IL-1*β* and IL-8 compared to *β*-actin through qPCR. (c, d) mRNA and protein expression levels of Bax/Bcl2 ratio compared to *β*-actin through qPCR and western blot. (e) Cell apoptosis rate of EPO treatment groups. Data are presented as mean ± SD (*n* = 3); ^∗^*P* < 0.05, ^∗∗^*P* < 0.01, ^∗∗∗^*P* < 0.001, and ^∗∗∗∗^*P* < 0.0001.

Cell viability suggested that EPO could rescue the proliferation of inflammatory PDLSCs on days 3, 5, and 7, especially in 20 IU/mL groups (Figure 4(a)). The mRNA expression level of IL-1*β* and IL-8 downregulated under the treatment of EPO (Figure 4(b)).

Under EPO treatment, qPCR and western blot revealed that the Bax/Bcl2 ratio was downregulated, indicating a declining trend of cell apoptosis (Figures 4(c) and 4(d)). The declining trend could also be observed in cell apoptosis assay (Figure 4(e)). It was concluded that EPO attuned the inflammation and cell apoptosis raised by TNF-*α*.

### 3.5. EPO Promoted Autophagy and Angiogenesis of PDLSCs under Inflammatory Microenvironment

**Figure 5:**
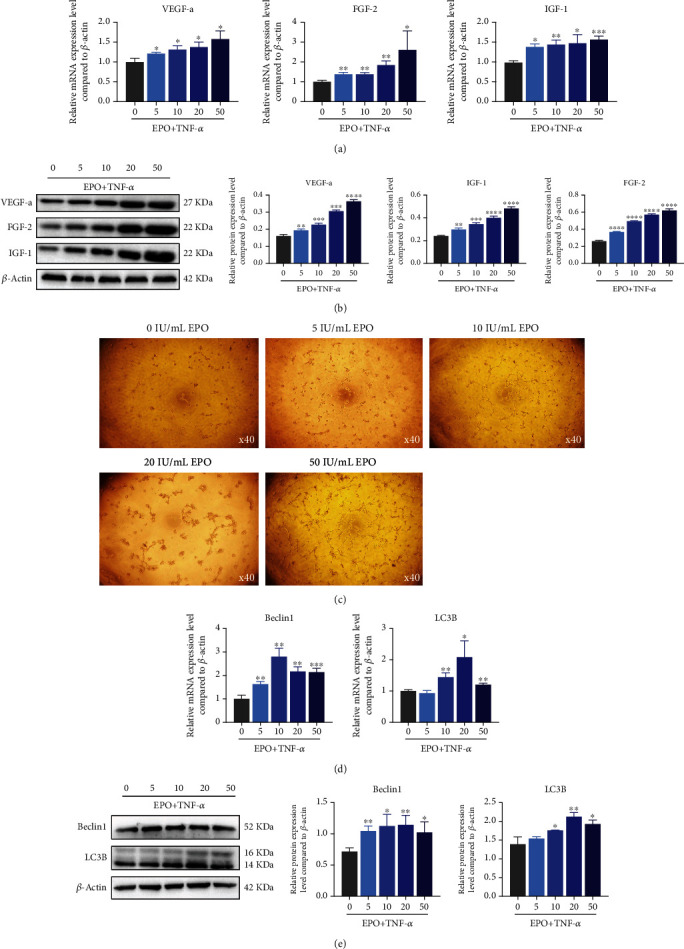
EPO promoted autophagy and angiogenesis of PDLSCs under inflammatory microenvironment. (a, b) mRNA and protein expression levels of VEGF-a, FGF-2, and IGF-1 compared to *β*-actin through qPCR and western blot. (c) Tube formation under EPO treatment. (d, e) mRNA and protein expression levels of Beclin1 and LC3B compared to *β*-actin through qPCR and western blot. Data are presented as mean ± SD (*n* = 3); ^∗^*P* < 0.05, ^∗∗^*P* < 0.01, ^∗∗∗^*P* < 0.001, and ^∗∗∗∗^*P* < 0.0001.

According to the mRNA/protein expression trend, EPO improved the expression of VEGF-a, IGF-1, and FGF-2 in a concentration-dependent manner (Figures 5(a) and 5(b)). Tube formation in vitro exhibited that EPO contributed to the angiogenic capacity of PDLSCs especially in 10, 20, and 50 IU/mL groups (Figure 5(c)).

Autophagy depressed by TNF-*α* was also promoted by EPO, based on the results of the mRNA/protein expression trend of Beclin1 and LC3B (Figures 5(d) and 5(e)).

### 3.6. EPO Regulated Autophagy, Apoptosis, and Angiogenesis of PDLSCs through the Akt/ERK1-2/BAD Signaling Pathway under Inflammatory Microenvironment

**Figure 6:**
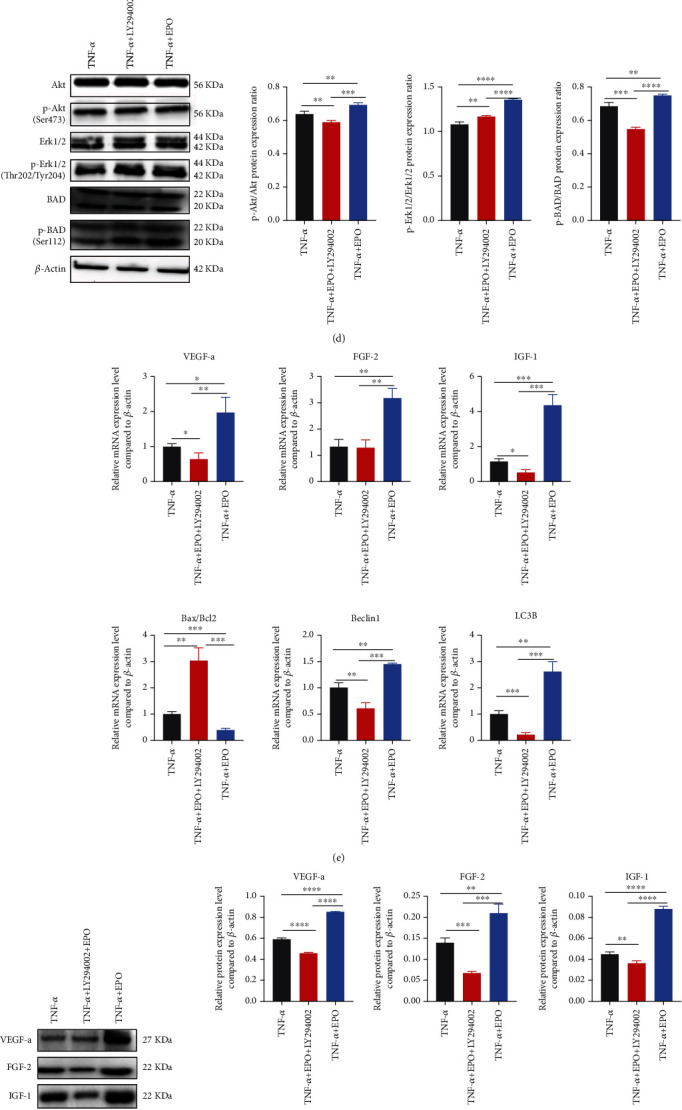
EPO regulated autophagy, apoptosis, and angiogenesis of PDLSCs through the Akt/ERK1-2/BAD signaling pathway under inflammatory microenvironment. (a) Bubble map of KEGG enrichment of RNA sequencing. (b, c) Heat map of enriched genes and qPCR validation (CSF3, ITGA10, PCK2, THBS4, DUSP4, ERGE, and KDR). (d) Protein expression levels of Akt, p-Akt, Erk1/2, p-Erk1/2, BAD, and p-BAD through western blot. (e, f) mRNA and protein expression levels of VEGF-a, FGF-2, IGF-1, Bax/Bcl2, Beclin1, and LC3B through qPCR and western blot. (g) Tube formation under different treatments. (h) Cell apoptosis rate under different treatments. (i, j) Immunofluorescence on DAPI, cytoskeleton, and VEGF-a/LC3B. Data are presented as mean ± SD (*n* = 3); ^∗^*P* < 0.05, ^∗∗^*P* < 0.01, ^∗∗∗^*P* < 0.001, and ^∗∗∗∗^*P* < 0.0001.

RNA sequencing was conducted to explore the signaling pathway aroused by EPO, recommending significant upregulation of the Akt signaling pathway (Figure 6(a)). To test its validation and reliability, qPCR was used to compare mRNA changing trend, affirming the result of RNA sequencing (Figures 6(b) and 6(c)). Through searching for the KEGG maps, some crucial regulatory factors of the P13K/Akt signaling pathway (Akt, ERK1/2, and BAD) were focused and phosphorylation levels were measured by western blot (Figure 6(d)). And phosphorylation levels could be depleted by LY294002, a specific inhibitor of the P13K/Akt signaling pathway. qPCR and western blot demonstrated that LY294002 could decrease effects of EPO on cell autophagy, apoptosis, and angiogenesis (Figures 6(e) and 6(f)). Matrigel plug showed that tube numbers increased in the TNF-*α*+EPO group (Figure 6(g)). And cell apoptosis decreased mostly in the TNF-*α*+EPO group (Figure 6(h)). Images of immunofluorescence (VEGF-a, LC3B) were in accordance with the results of western blot (Figures 6(i) and 6(j)).

### 3.7. EPO Moderated Apoptosis and Angiogenesis of PDLSCs through Targeting Autophagy under Inflammatory Microenvironment

**Figure 7:**
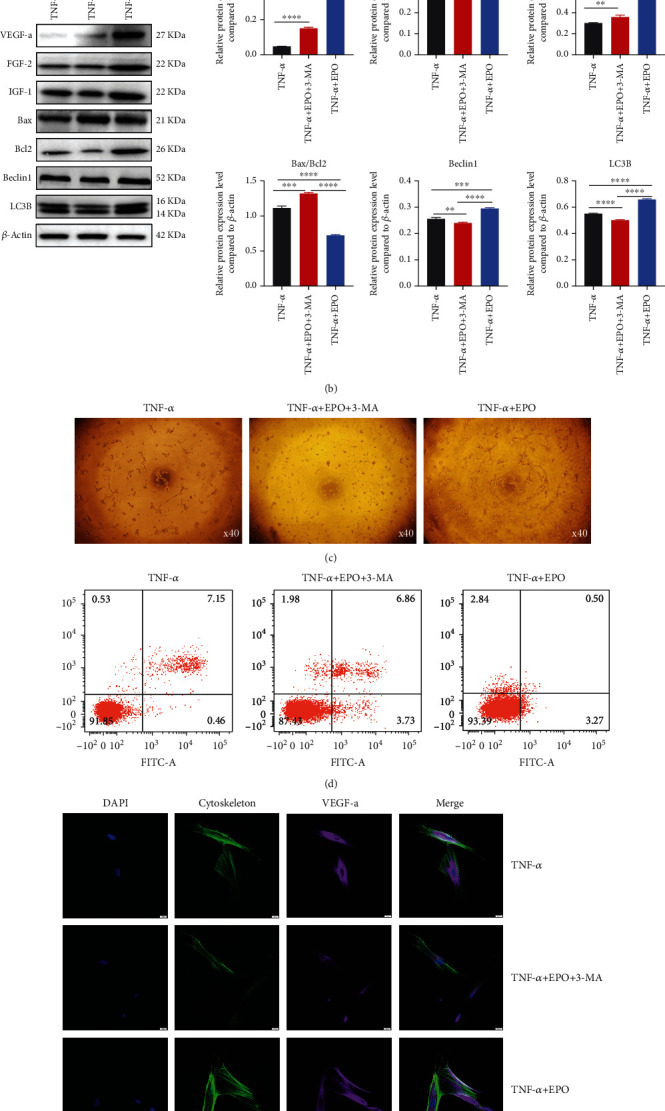
EPO moderated apoptosis and angiogenesis of PDLSCs through targeting autophagy under inflammatory microenvironment. (a, b) mRNA and protein expression levels of VEGF-a, FGF-2, IGF-1, Bax/Bcl2, Beclin1, and LC3B through qPCR and western blot. (c) Tube formation under different treatments. (d) Cell apoptosis rate under different treatments. (e, f) Immunofluorescence on DAPI, cytoskeleton, and VEGF-a/LC3B. Data are presented as mean ± SD (*n* = 3); ^∗^*P* < 0.05, ^∗∗^*P* < 0.01, ^∗∗∗^*P* < 0.001, and ^∗∗∗∗^*P* < 0.0001.

As an autophagy inhibitor, 3-MA was added to investigate how EPO regulated cell autophagy on apoptosis and angiogenesis. Both qPCR and western blot inferred that 3-MA could downregulate cell autophagy, further changing antiapoptosis and angiogenesis induced by EPO (Figures 7(a) and 7(b)). Matrigel plug showed that tube numbers increased in the TNF-*α*+EPO group (Figure 7(c)). And cell apoptosis decreased mostly in the TNF-*α*+EPO group (Figure 7(d)). Results of immunofluorescence (VEGF-a, LC3B) were in concord with western blot (Figures 7(e) and 7(f)).

## 4. Discussion

Periodontitis is always triggered by dental bacterial plaque and accelerated by local or wholesome factors. Regarding the pathological process, it involves the invasion of bacteria, activation of immune reaction, recession of junctional epithelium, and depredation of alveolar bone [35]. To defend harmful LPS originated from bacteria, TNF-*α* is excessively expressed in the process of periodontitis, which degrades periodontal tissue and fastens cell apoptosis [36, 37]. TNF-*α* is the core inflammatory cytokine during periodontitis, which is suitable for establishment of inflammatory microenvironment [38–40]. Biological behaviors of PDLSCs were always undermined under such inflammatory microenvironment [41–44].

Here, we selected TNF-*α* to mock the microenvironment of periodontitis, and we established a TNF-*α* concentration gradient to explore biological behaviors of inflammatory PDLSCs. Coherent with existed literatures [41, 45, 46], TNF-*α* inhibited cell viability and increased expression of inflammatory genes *via* the NF-*κ*B signaling pathway. Fang et al. [47] and Meng et al. [41] mentioned that TNF-*α* could also induce apoptosis and oxidative stress of PDLSCs, which was analogous to our study. In our research, TNF-*α* agitated the expression of IL-1*β* and IL-8, suppressed cell proliferation, and enforced Bax/Bcl2 expression ratio, especially in 50 ng/mL and 100 ng/mL groups. And other researchers also used 20 ng/mL or 50 ng/mL TNF-*α* to mimic the inflammatory microenvironment, which supported results. As the same, those also reckoned that proliferation rate was evidently suppressed and inflammatory cytokines expressed most on 72 hours [46, 48, 49].

Autophagy is reckoned as a double-edged sword for biological behaviors. In some views, autophagy played a harmful role in the pathogenesis, which indicated that autophagy was positively relevant to inflammatory level and apoptosis [50, 51], while some viewpoints displayed its potential therapeutic value, which could protect cells from apoptosis and promote vascularization [52]. It is controversial that TNF-*α* would decrease or increase cellular autophagy. Some researchers pointed that TNF-*α* contributed to autophagy to protect PDLSCs from apoptosis at an early stage, while attenuating autophagy in a long run [25]. Chen et.al [53] held that TNF-*α* often downregulated LC3B, Beclin1, and Atg7, resulting the osteogenic decline of PDLSCs. According to the results, the expression level of Beclin1 and LC3B was further diminished with the increasing concentration of TNF-*α*, which aggravated apoptosis. As the same, the secretions of VEGF-a, FGF-2, and IGF-1 were declined with the increasing concentration of TNF-*α*, which denoted the decreasing angiogenic level. Taken together, autophagy impaired by TNF-*α* was considered as a protective factor for PDLSCs in the inflammatory microenvironment. Collectively, 50 ng/mL TNF-*α* was picked for the continuing experiment.

EPO has been learned as a multifunctional cytokine/drug for wound healing and bone regeneration, particularly in the realm of periodontology [54–56]. An avalanche of researches argued that EPO attenuated inflammation, contributed to antiapoptosis, enhanced autophagy, and promoted angiogenesis [13, 18, 57–59]. Meanwhile, some researches [60–62] supported that EPO receptor (EPOR) was expressed in many CD105^+^/CD90^+^/CD44^+^ stem cells—such as PDLSCs, dental pulp stem cells, and bone marrow progenitor cells—which demonstrated that the EPO/EPOR signaling pathway played a crucial role in regulating biological behaviors of these cells. And PDLSCs were also characterized as CD105^+^, CD90^+^, and CD44^+^ mesenchymal stem cells. The expression of EPOR on CD105^+^/CD90^+^/CD44^+^ PDLSCs provided basics for EPO treatment. Therefore, we conducted experiments focusing on the protective effects of EPO for PDLSCs. In the inflammatory microenvironment, proliferation of PDLSCs was enhanced with the treatment of EPO, especially in the 20 IU/mL group. IL-1*β* and IL-8 were obviously resisted after the treatment of EPO. Likewise, a similar experiment showed that the inflammation level of PDLSCs could be attenuated by ascorbic acid, revealing that inflammation of PDLSCs was significantly related with the NF-*κ*B signaling pathway and DNMT1, which could activate expression of proinflammatory cytokines [63]. The expression ratio of Bax/Bcl2 was also inhibited, which protected cells from early or late apoptosis. Additionally, the strengthening expression of VEGF-a, FGF-2, and IGF-1 could also be noted under the treatment of EPO, which indicated the upregulating angiogenesis of PDLSCs. Autophagy-related genes Beclin1 and LC3B were also heightened by EPO, especially in 10 IU/mL and 20 IU/mL groups. Above these data, it was speculated that EPO could preserve antiapoptosis, angiogenesis, and autophagy of PDLSCs under inflammatory microenvironment, which suggested its promising use for controlling periodontitis. Regarding its various effects on PDLSCs, therefore, 20 IU/mL EPO was the optimal group for subsequent experiments.

The Akt signaling pathway always participated in the regulation of apoptosis, autophagy, and angiogenesis [27, 29]. Akt was also the main target of EPO, bridging the downstream signal and interacting with autophagy and then stirring up biological activities and determining cell fate [58, 59, 64]. According to RNA-sequencing results, KEGG enrichment demonstrated that the P13K/Akt signaling pathway was significantly upregulated in EPO treatment groups. Through analyzing the KEGG map, Akt, Erk1/2, and BAD were taken into measurement. According to the existing references, LY294002, an inhibitor for the PI3K/Akt signaling pathway, has been confirmed that could also inhibit the phosphorylation of Akt, ERK1/2, and BAD in human or rat cells [65–67]. Phosphorylated Akt, Erk1/2, and BAD protein were raised by EPO and suppressed by TNF-*α* or PI3K/Akt specific inhibitor—LY294002, denoting that the Akt/Erk1/2/BAD signaling pathway was activated through phosphorylation. p-Erk1/2 and p-BAD changed following the change of phosphorylated Akt. VEGF-a, FGF-2, and IGF-1 levels may be regulated by p-Erk1/2 [68]; Bax/Bcl2 ratio may be relevant to p-BAD [69]; Beclin1 and LC3B may be targeted by Akt [70]. Additionally, some researchers also found that expression of LC3 and Erk/p-Erk played a vital role in the regulation of dental pulp stem cell inflammation, suggesting that the prompt LC3 and p-Erk rescued autophagy, which was consistent with our results [71]. LY294002 could reverse tube numbers induced by EPO and also aggravate cell apoptosis attenuated by EPO. Collectively, EPO could enhance antiapoptosis, angiogenesis, and autophagy of PDLSCs via the Akt/Erk1/2/BAD signaling pathway under inflammatory microenvironment, which would be blocked by LY294002.

Autophagy also dually moderates apoptosis and angiogenesis [24, 25]. In one viewpoint, autophagy maintains cell survivability through blocking the Akt signaling pathway [72, 73]. Contradictorily, another view argued that autophagy was reserved by activating the Akt signaling pathway [28, 30]. Our data has revealed that autophagy could be regulated by the Akt/Erk1/2/BAD signaling pathway. Wondering whether autophagy increased by EPO could regulate apoptosis and angiogenesis, autophagy inhibitor 3-methyladenine (3-MA) was used to verify its effects. When 3-MA was added, Beclin1, LC3B, VEGF-a, FGF-2, and IGF-1 levels decreased compared to the TNF-*α*+EPO group; Bax/Bcl2 ratio increased compared to the TNF-*α*+EPO group. Noticeably, 3-MA reduced tube numbers enhanced by EPO and augmented cell apoptosis rescued by EPO. Comprehensively, autophagy lifted by EPO could also moderate apoptosis and angiogenesis of PDLSCs under inflammatory microenvironment.

However, there still exists drawback, such as lack of animal experiments. A step further, *in vivo* experiments would be conducted to testify its *in vivo* effects. Due to the limitations of fundamental experiment, clinical performance was not certain, requiring further research.

Above all, EPO attenuated inflammation, reduced apoptosis, rescued autophagy, and augmented angiogenesis of PDLSCs under inflammatory microenvironment. And its potential mechanism was also conducted. EPO activated autophagy to moderate apoptosis and angiogenesis via the Akt/Erk1/2/BAD signaling pathway (Supplementary Figure 2, graphical abstract). Our research provided a novel strategy for curing periodontal inflammation and accomplishing angiogenic tissue engineering.

## 5. Conclusion

It could be demonstrated that EPO could protect biological behaviors of PDLSCs from inflammatory microenvironment and promote angiogenic tissue regeneration, which brought a brand-new sight for periodontal tissue engineering.

## Figures and Tables

**Table 1:** Primer sequences of target genes for qPCR.

Target gene name (human)	Forward primer sequence	Reverse primer sequence
IL-1*β*	5′-ACAGATGAAGTGCTCCTTCCA-3′	5′-GTCGGAGATTCGTAGCTGGAT-3′
IL-8	5′-ATGACTTCCAAGCTGGCCGTGGCT-3′	5′-TCTCAGCCCTCTTCAAAAACTTCTC-3′
Bax	5′-GATGCGTCCACCAAGAAGCTGAG-3′	5′-CACGGCGGCAATCATCCTCTG-3′
Bcl2	5′-TGGACTGCCCCAGAAAAATA-3′	5′-TCTTGATTGAGCGAGCCTTT-3′
VEGF-a	5′-CATGCAGATTATGCGGATCAA-3′	5′-GCATTCACATTTGTTGTGCTGTAG-3′
FGF-2	5′-AAGAGCGACCCTCACATCAAG-3′	5′-GTTCGTTTCAGTGCCACATACC-3′
IGF-1	5′-TGTCCTCCTCGCATCTCTTCT-3′	5′-CCATACCCTGTGGGCTTGT-3′
Beclin1	5′-ATTCGAGAGCAGCATCC AAC-3′	5′-AACAGGAAGCTGCTTCTCAC-3′
LC3B	5′-GGGGCCTCGGAGCAAGTCCA-3′	5′-CCCCGGGAGCCTCGTTCAGGT-3′
DUSP4	5′-TACTCGGCGGTCATCGTCTACG-3′	5′-CGGAGGAAAACCTCTCATAGCC-3′
EREG	5′-GGACAGACTTCCAAGATGAGCC-3′	5′-CCACACTGCATTCATCAGGAGAG-3′
KDR	5′-GGAACCTCACTATCCGCAGAGT-3′	5′-CCAAGTTCGTCTTTTCCTGGGC-3′
ITGA10	5′-CCTTTGCTTCCAAGTGACCTCC-3′	5′-CAGAGCCATCAAATGCTGCACG-3′
CSF3	5′-TCCAGGAGAAGCTGGTGAGTGA-3′	5′-CGCTATGGAGTTGGCTCAAGCA-3′
PCK2	5′-TAGTGCCTGTGGCAAGACCAAC-3′	5′-GAAGCCGTTCTCAGGGTTGATG-3′
THBS4	5′-ACCGACAGTAGAGATGGCTTCC-3′	5′-CGTCACATCTGAAGCCAGGAGA-3′
*β*-Actin	5′-CCTGGCACCCAGCACAAT-3′	5′-GCCGATCCACACGGAGTA-3′

**Table 2:** Primary antibodies for western blot and immunofluorescence.

Primary antibody	Source	Diluted multiple
Anti-Bax	Abcam, ab182733	1 : 2000
Anti-Bcl2	Abcam, ab182858	1 : 2000
Anti-VEGF-a	Abcam, ab185238	1 : 00000
Anti-FGF-2	Abcam, ab208687	1 : 1000
Anti-IGF-1	Abcam, ab133542	1 : 1000
Anti-Beclin1	Abcam, ab210498	1 : 1000
Anti-LC3B	Abcam, ab192890	1 : 2000
Anti-Akt	CST, #4691	1 : 1000
Anti-p-Akt (Ser473)	Abcam, ab81283	1 : 5000
Anti-Erk1/2	CST, #4695	1 : 1000
Anti-p-Erk1/2 (Thr202/Tyr204)	CST, #4370	1 : 2000
Anti-BAD	CST, #9292	1 : 1000
Anti-p-BAD (Ser112)	Abcam, ab129192	1 : 5000
Anti-*β*-actin	CST, #4970	1 : 1000

## Data Availability

All the research data used to support the findings of this study are available from the corresponding author upon request.

## Abbreviations

EPO: Erythropoietin
PDLSCs: Periodontal ligament stem cells
TNF-*α*: Tumor necrosis factor-*α*
APSCs: Apical papilla stem cells
MSCs: Mesenchymal stem cells
BMSCs: Bone marrow stem cells
VECs: Vein endothelial cells
TNFAIP3: Tumor necrosis factor alpha-induced protein 3
PDLCs: Periodontal ligament cells
LPS: Lipopolysaccharide
ASCs: Adipose-derived stem cells
FBS: Fetal bovine serum
CCK-8: Cell Counting Kit-8
qPCR: Real-time quantitative polymerase chain reaction.

## Supplementary Materials

Supplementary 1 Supplementary Figure 1: results of western blot assay have been replicated for three times and blots are included in the file.

Supplementary 2 Supplementary Figure 2: EPO attenuated inflammation, reduced apoptosis, rescued autophagy, and augmented angiogenesis of PDLSCs under inflammatory microenvironment. EPO activated autophagy to moderate apoptosis and angiogenesis via the Akt/Erk1/2/BAD signaling pathway.
