# Supplementary material for: Erythropoietin Activates Autophagy to Regulate Apoptosis and Angiogenesis of Periodontal Ligament Stem Cells via the Akt/ERK1/2/BAD Signaling Pathway under Inflammatory Microenvironment
Source: Stem Cells Int. 2022 Sep 20;2022:9806887. doi: 10.1155/2022/9806887 (PMC9527112; doi:10.1155/2022/9806887)
Supplement: Supplementary 1 — Supplementary Figure 1: results of western blot assay have been replicated for three times and blots are included in the file. [file 9806887.f1.pdf]

Raw data of western blot

Figure 2

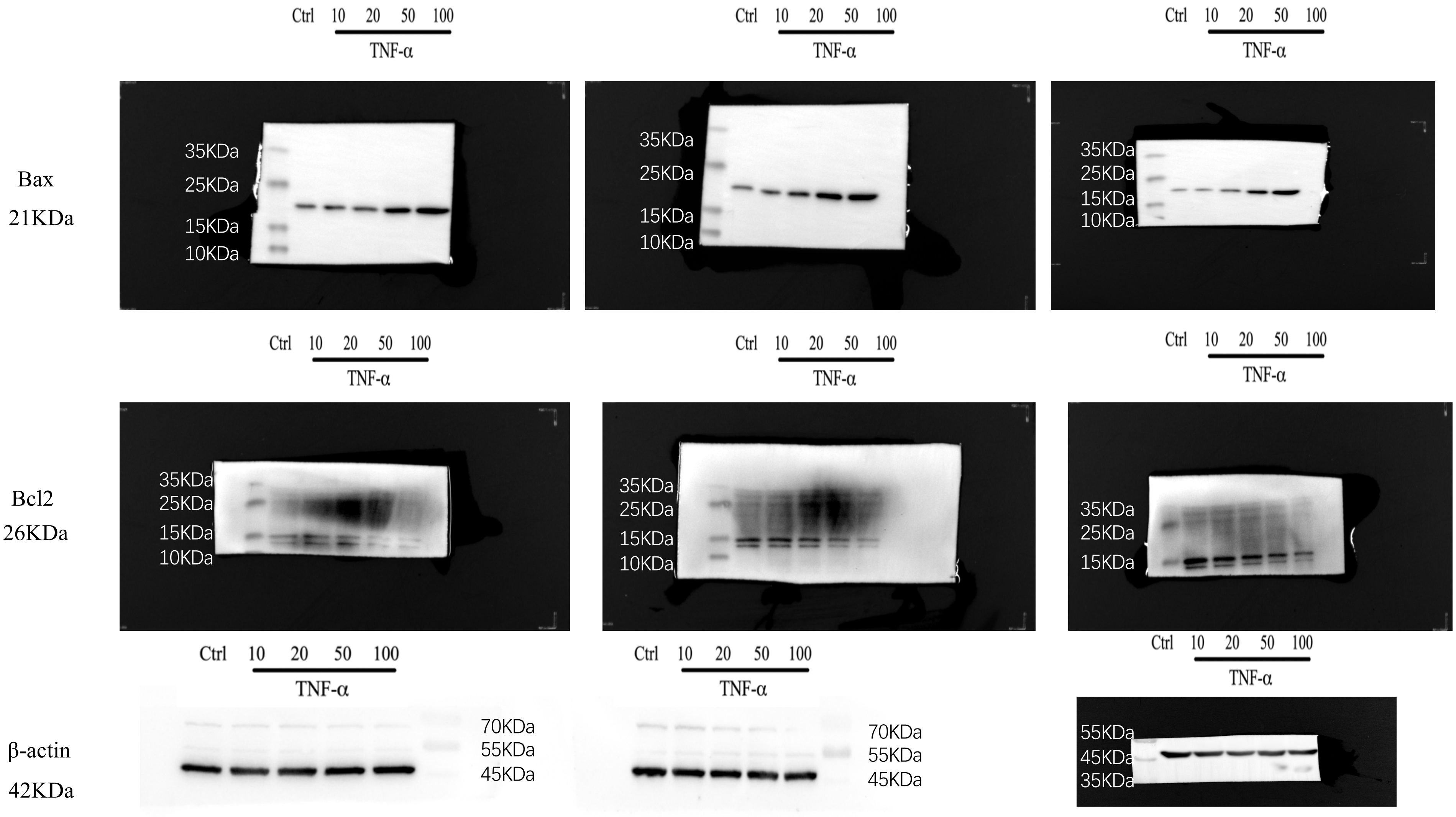
Ctrl 10 20 50 100
TNF-α
Bax
21KDa
35KDa
25KDa
15KDa
10KDa
Bcl2
26KDa
β-actin
42KDa
70KDa
55KDa
45KDa
35KDa

Figure 3

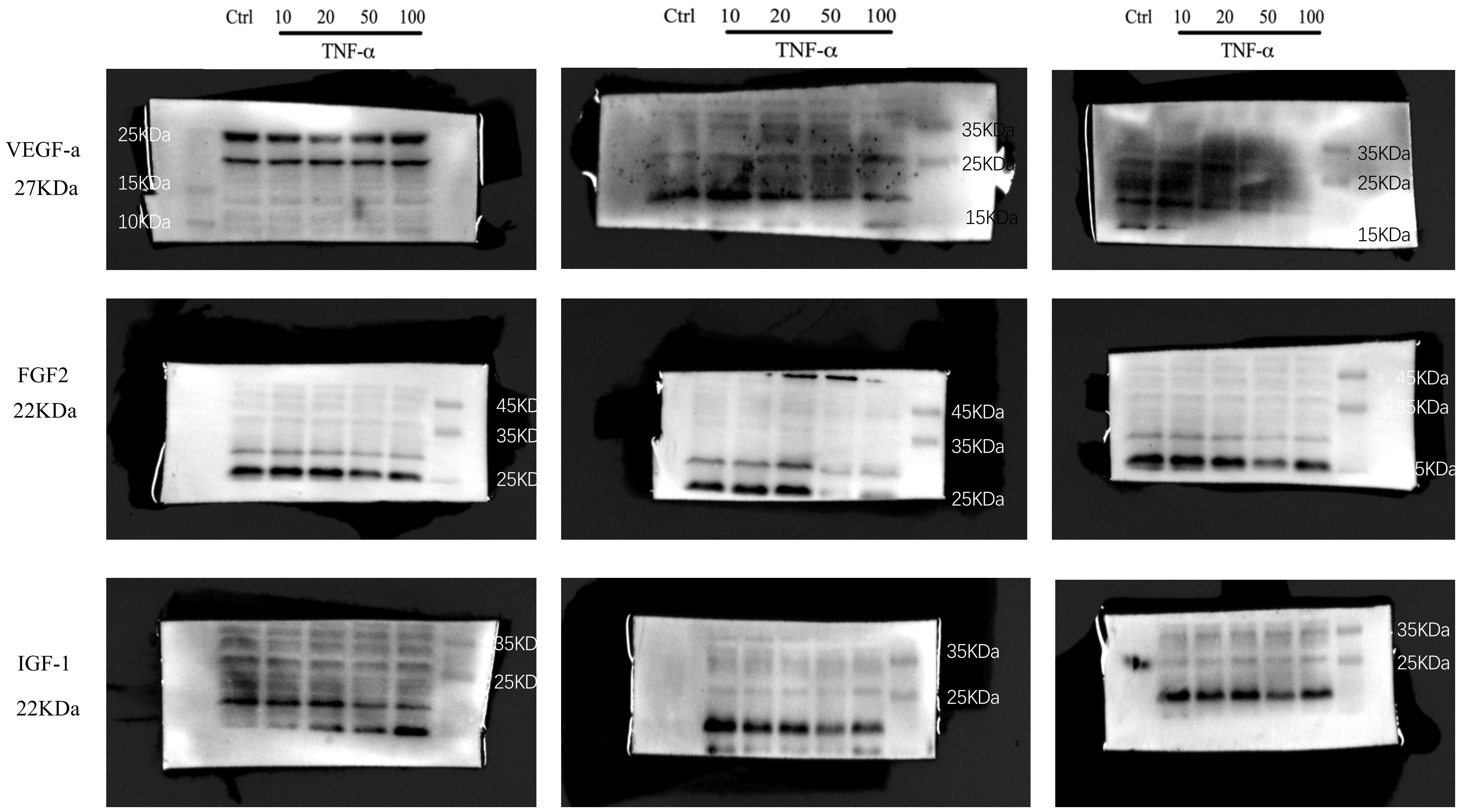

Ctrl 10 20 50 100
TNF-α
VEGF-a
27KDa
FGF2
22KDa
IGF-1
22KDa
25KDa
15KDa
10KDa
35KDa
45KDa

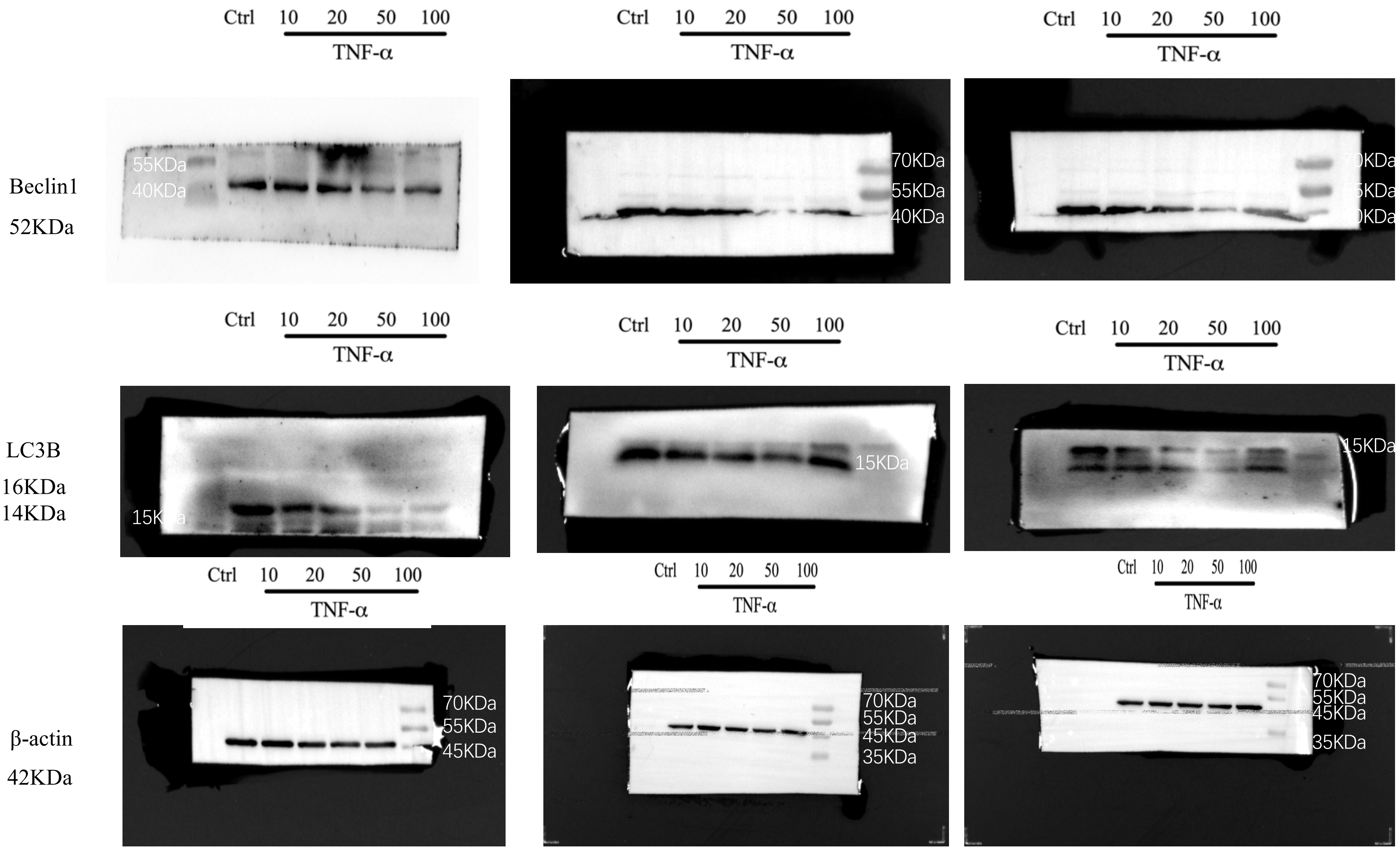
Ctrl
10
20
50
100
TNF-α
Beclin1
52KDa
55KDa
40KDa
70KDa
55KDa
40KDa
LC3B
16KDa
14KDa
15KDa
β-actin
42KDa
70KDa
55KDa
45KDa
35KDa

Figure 4

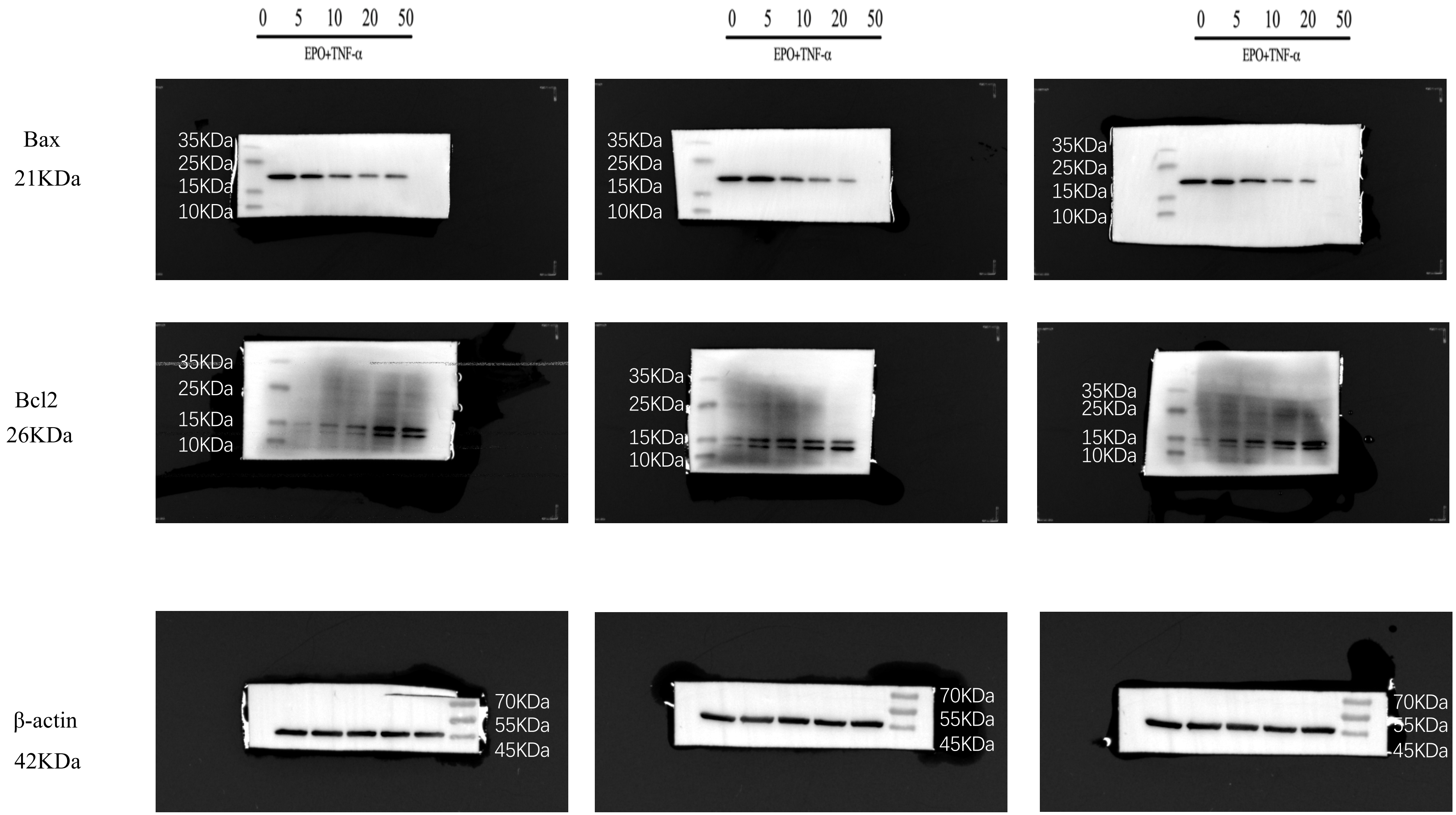
0 5 10 20 50
EPO+TNF-α
0 5 10 20 50
EPO+TNF-α
0 5 10 20 50
EPO+TNF-α
Bax
21KDa
Bcl2
26KDa
β-actin
42KDa
35KDa
25KDa
15KDa
10KDa
70KDa
55KDa
45KDa

Figure 5

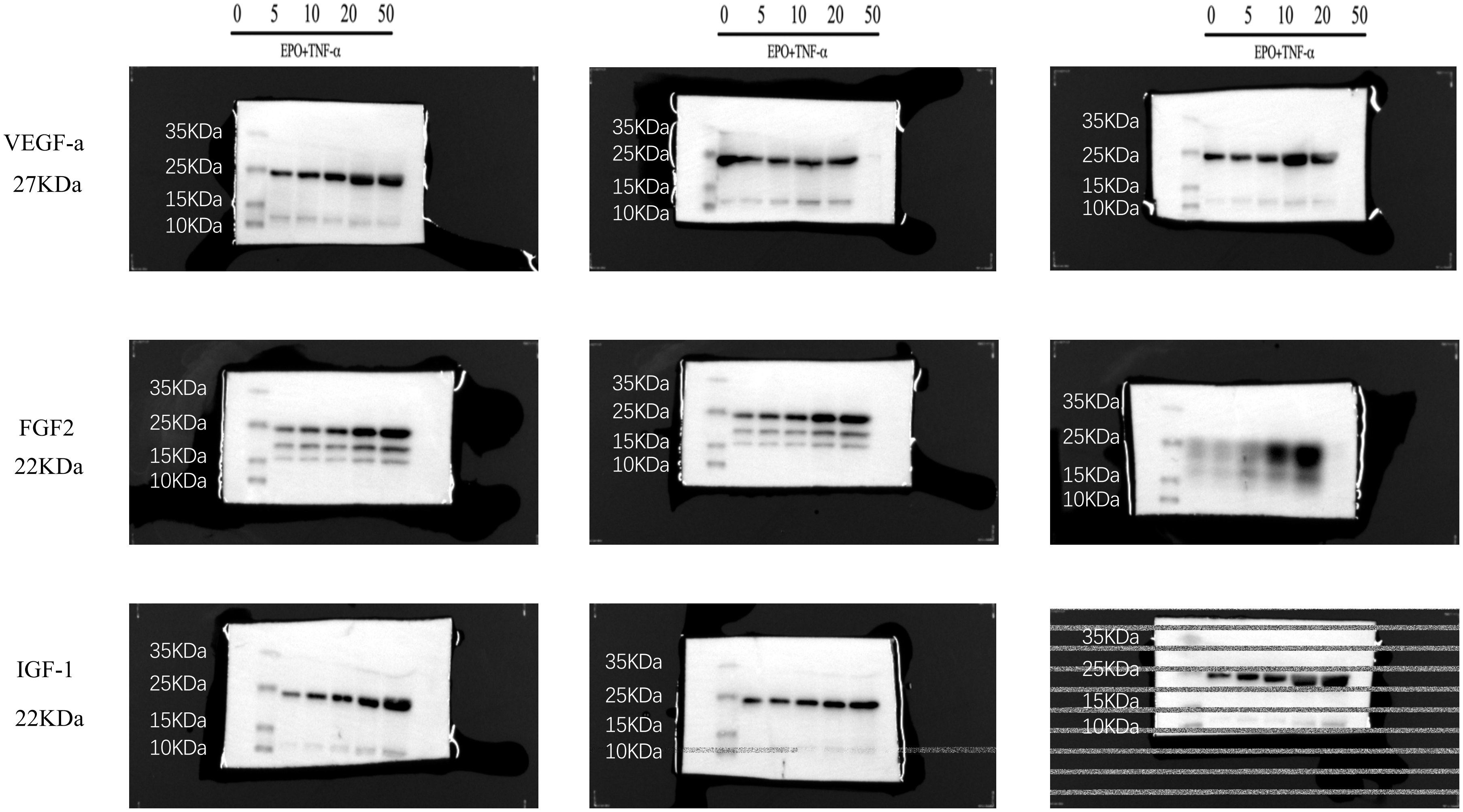
0 5 10 20 50
EPO+TNF-α
0 5 10 20 50
EPO+TNF-α
0 5 10 20 50
EPO+TNF-α
VEGF-a
27KDa
FGF2
22KDa
IGF-1
22KDa
35KDa
25KDa
15KDa
10KDa

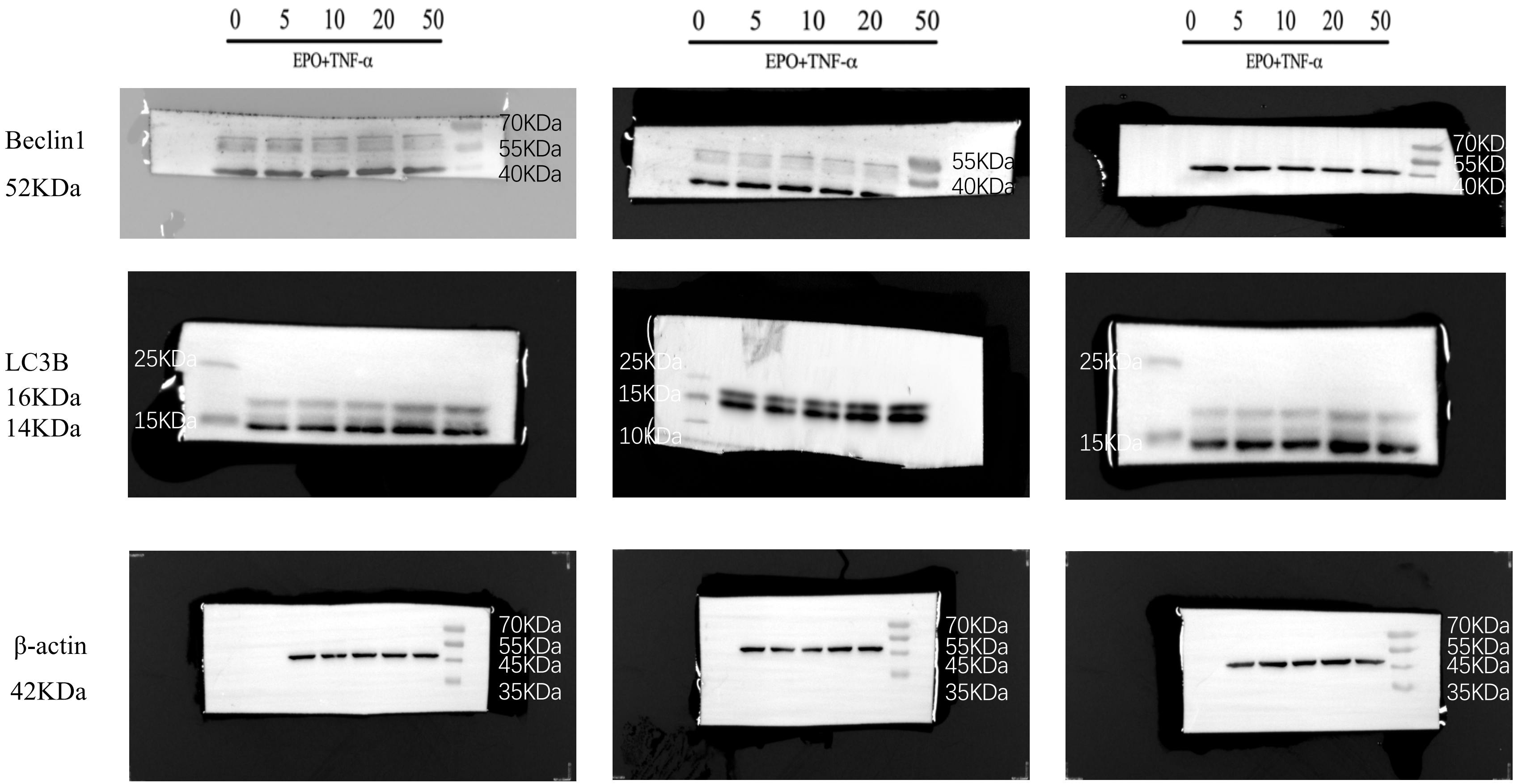
0 5 10 20 50
EPO+TNF-α
0 5 10 20 50
EPO+TNF-α
0 5 10 20 50
EPO+TNF-α
Beclin1
52KDa
70KDa
55KDa
40KDa
55KDa
40KDa
LC3B
16KDa
14KDa
25KDa
15KDa
25KDa
15KDa
10KDa
25KDa
15KDa
β-actin
42KDa
70KDa
55KDa
45KDa
35KDa
70KDa
55KDa
45KDa
35KDa
70KDa
55KDa
45KDa
35KDa

Figure 6

Akt

56KDa

p-Akt

56KDa

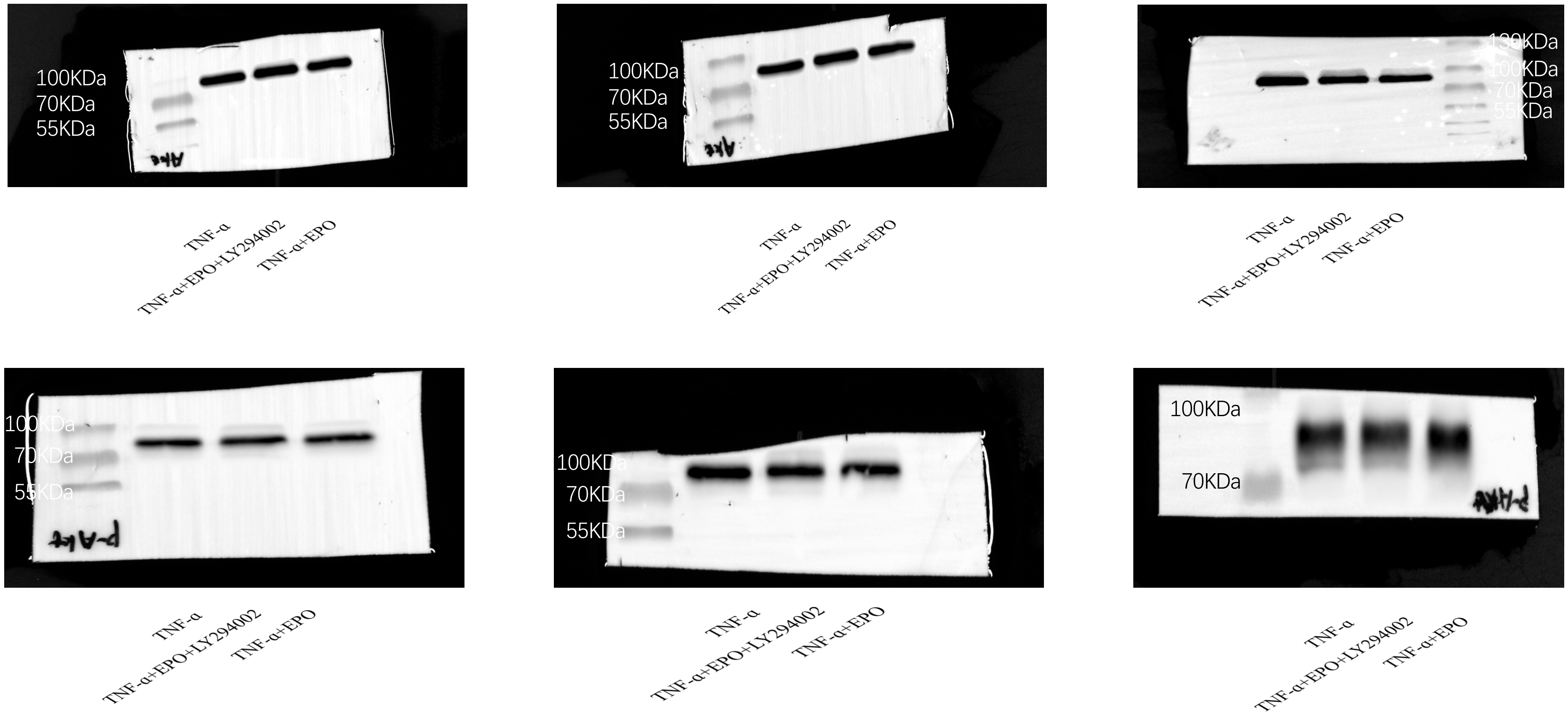

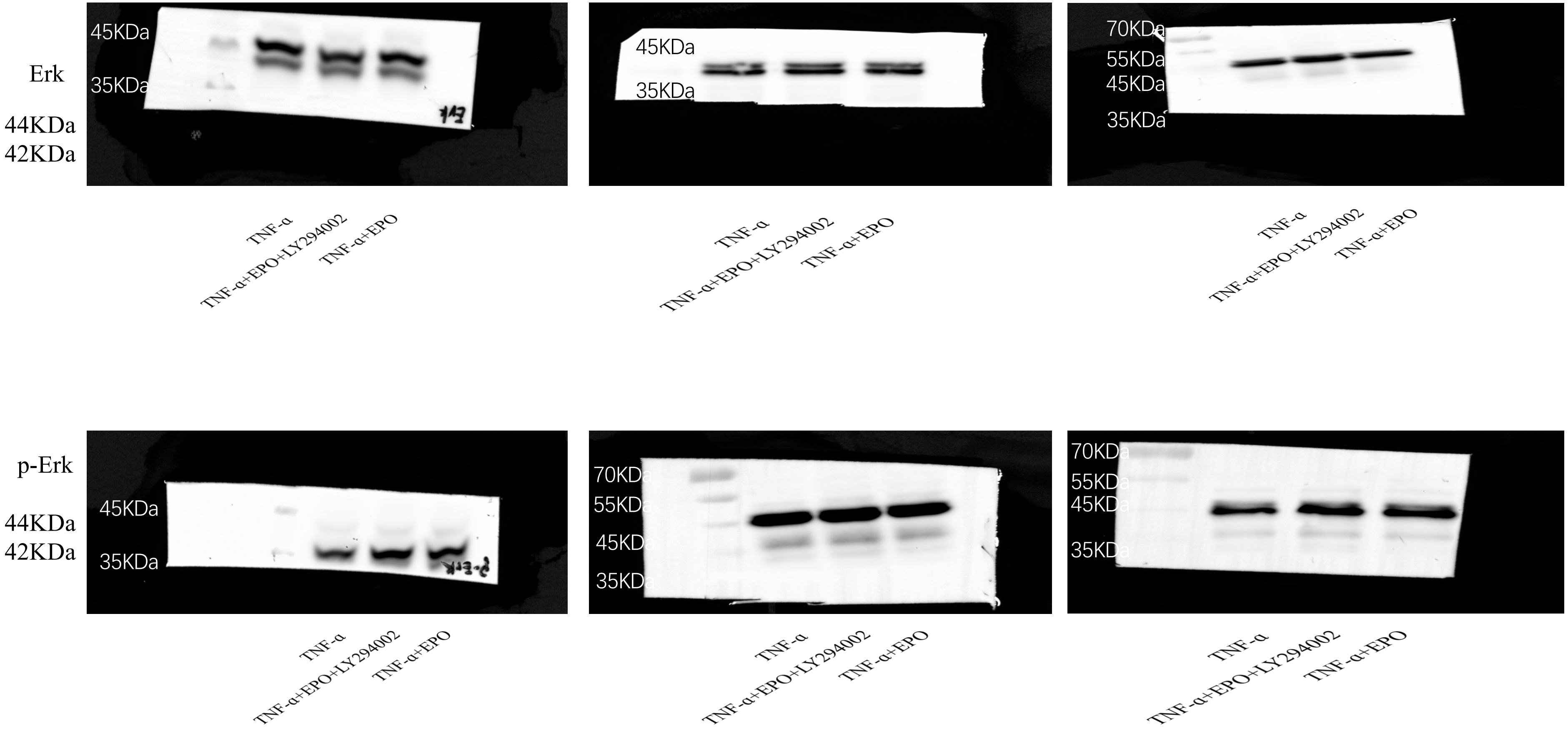
Erk
44KDa
42KDa
45KDa
35KDa
TNF-α
TNF-α+EPO+LY294002
TNF-α+EPO
45KDa
35KDa
TNF-α
TNF-α+EPO+LY294002
TNF-α+EPO
70KDa
55KDa
45KDa
35KDa
TNF-α
TNF-α+EPO+LY294002
TNF-α+EPO
p-Erk
44KDa
42KDa
45KDa
35KDa
TNF-α
TNF-α+EPO+LY294002
TNF-α+EPO
70KDa
55KDa
45KDa
35KDa
TNF-α
TNF-α+EPO+LY294002
TNF-α+EPO
70KDa
55KDa
45KDa
35KDa
TNF-α
TNF-α+EPO+LY294002
TNF-α+EPO

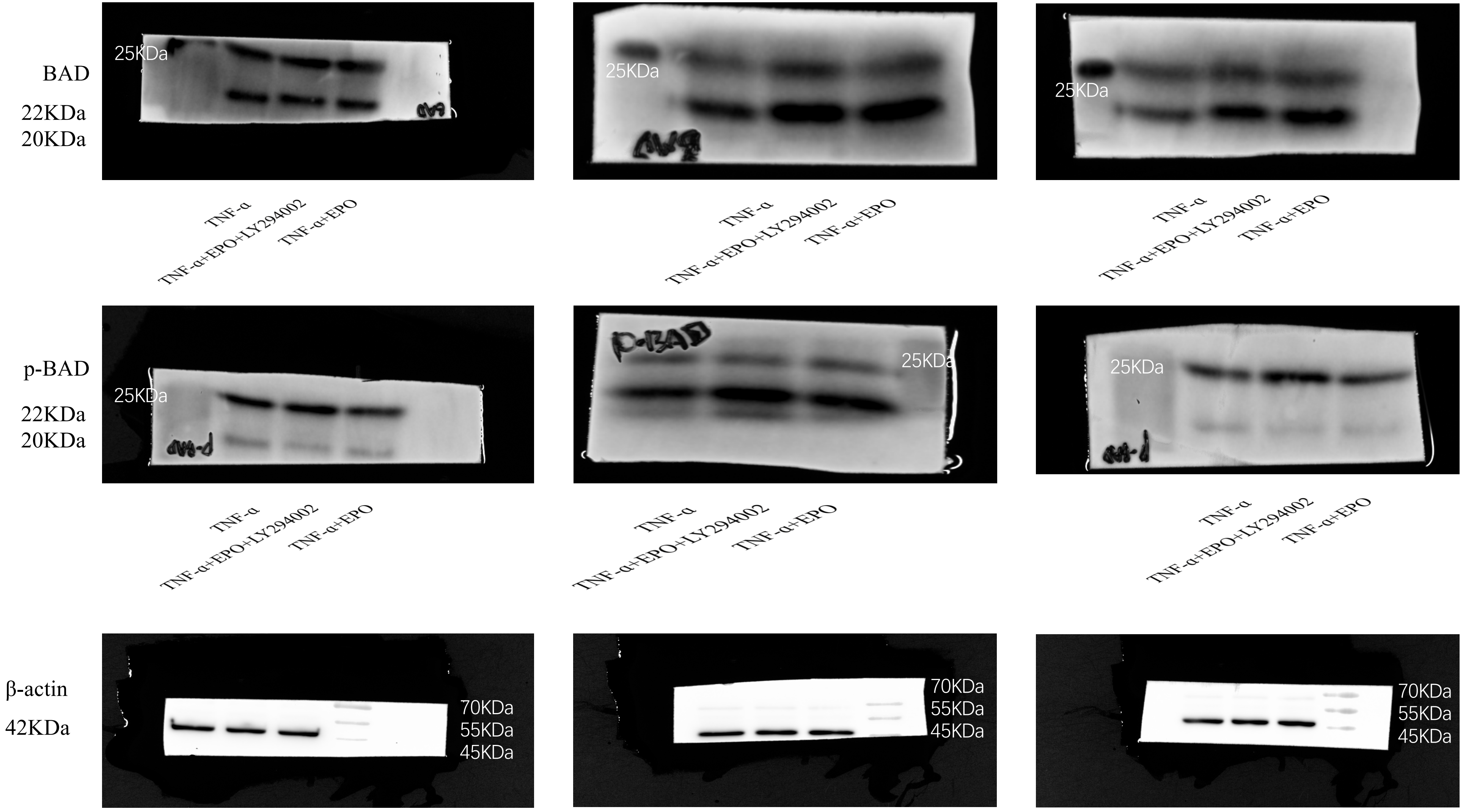
BAD
22KDa
20KDa
25KDa
TNF-α
TNF-α+EPO+LY294002
TNF-α+EPO
p-BAD
22KDa
20KDa
25KDa
TNF-α
TNF-α+EPO+LY294002
TNF-α+EPO
β-actin
42KDa
70KDa
55KDa
45KDa

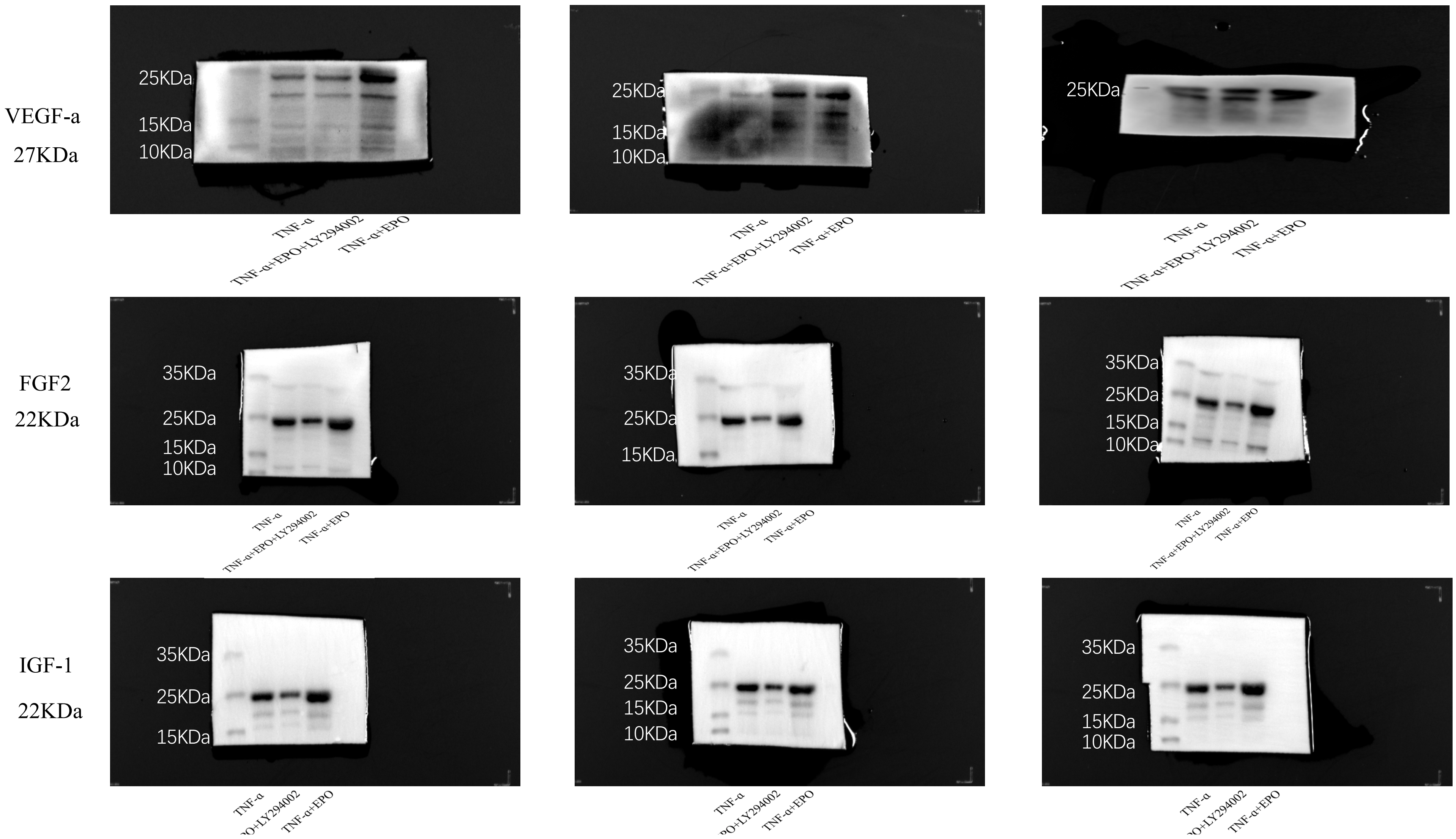
VEGF-a
27KDa
25KDa
15KDa
10KDa
TNF-α
TNF-α+EPO+LY294002
TNF-α+EPO
FGF2
22KDa
35KDa
25KDa
15KDa
10KDa
IGF-1
22KDa
35KDa
25KDa
15KDa
10KDa

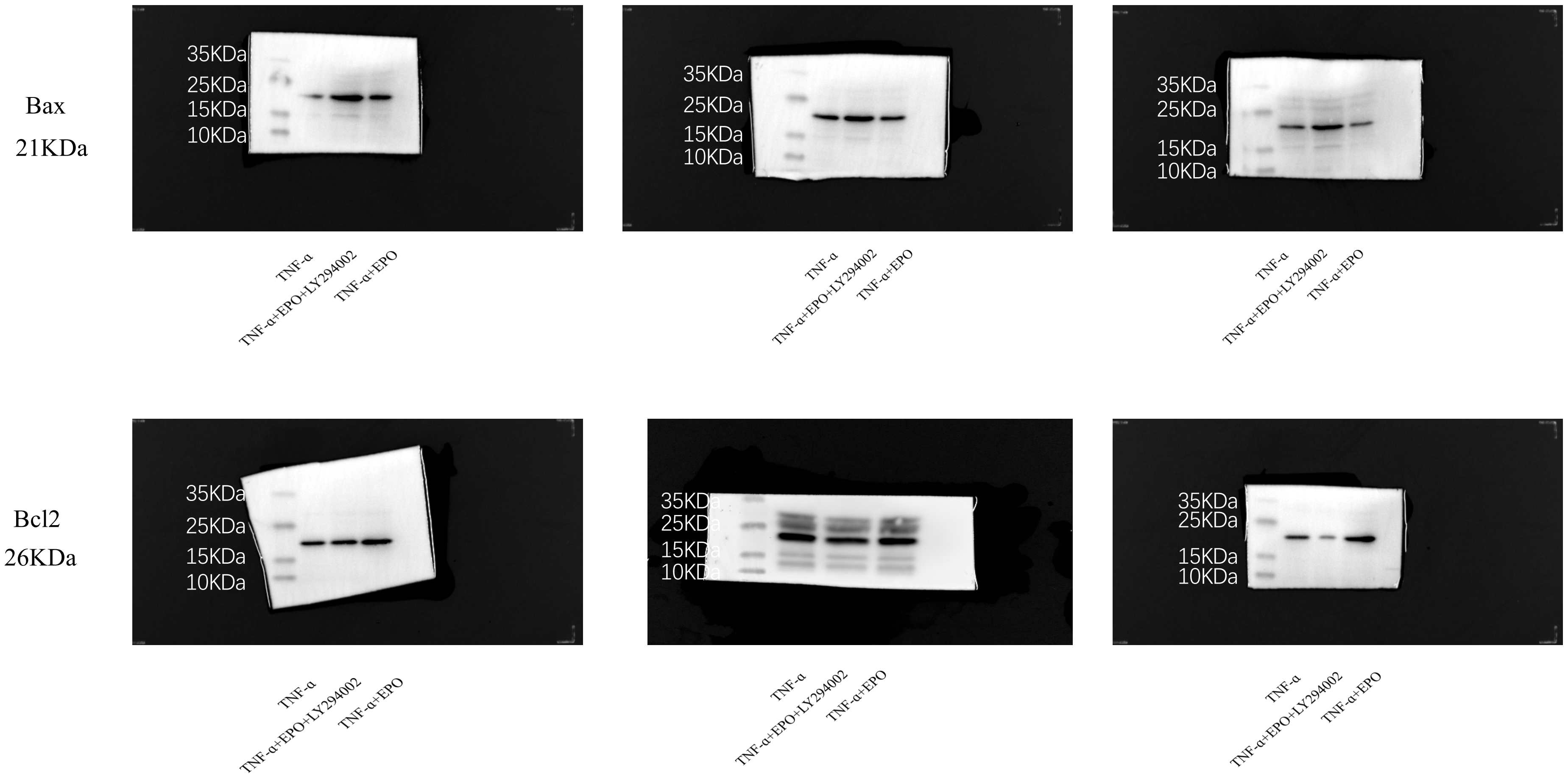
Bax
21KDa
35KDa
25KDa
15KDa
10KDa
TNF-α
TNF-α+EPO+LY294002
TNF-α+EPO
Bcl2
26KDa

Beclin1

52KDa

LC3B

16KDa

14KDa

β-actin

42KDa

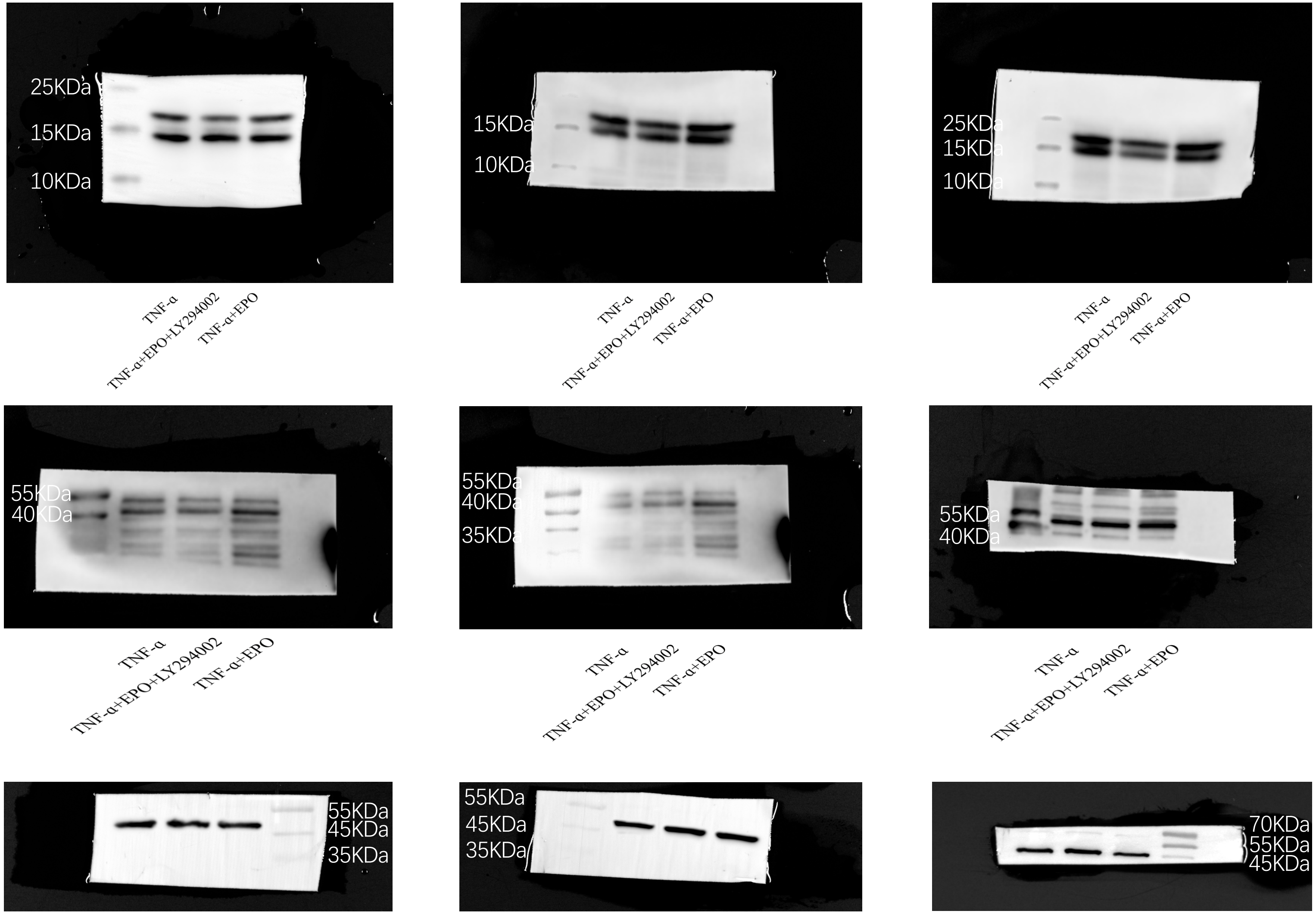

Figure7

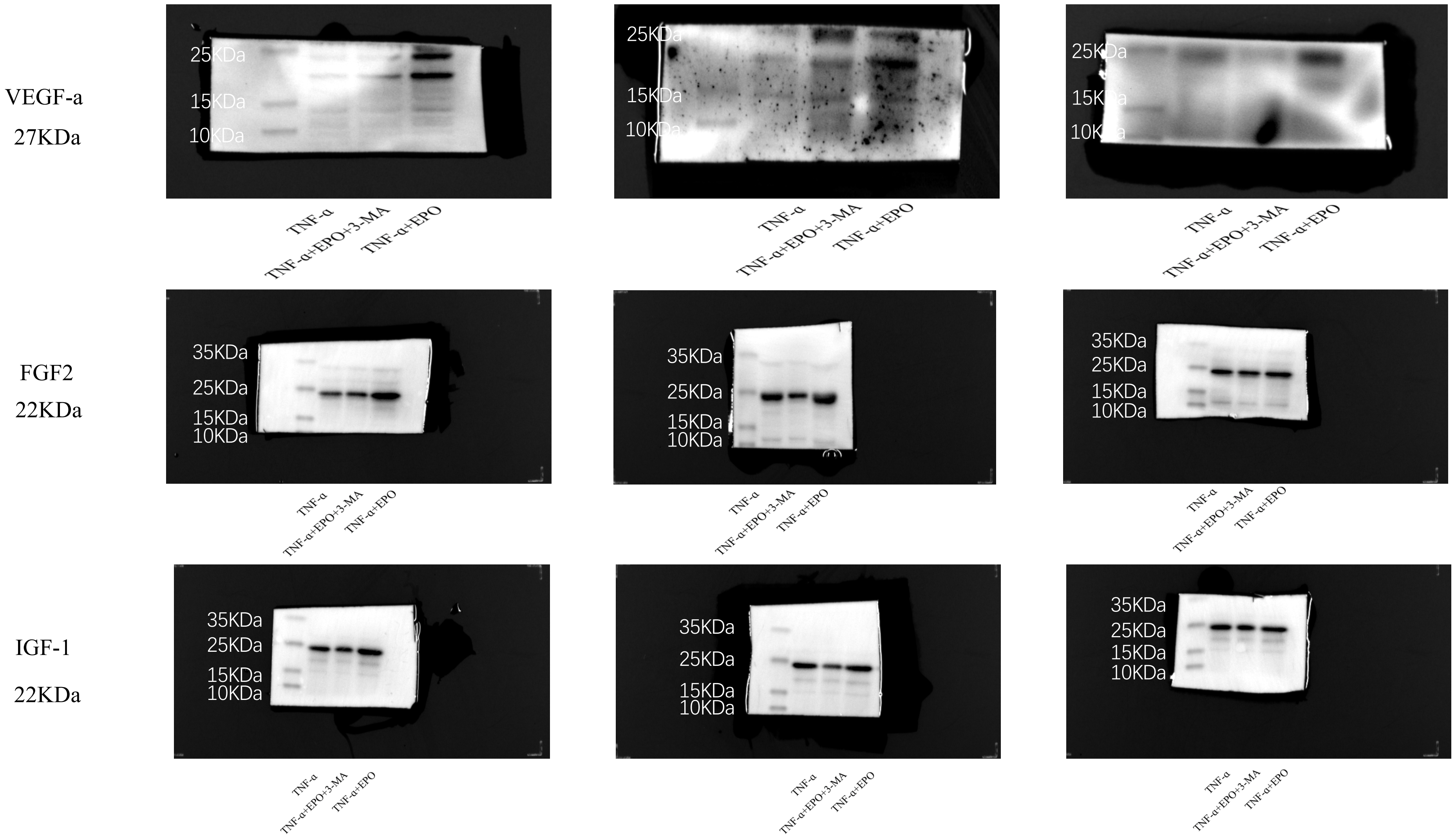
VEGF-a
27KDa
25KDa
15KDa
10KDa
TNF-α
TNF-α+EPO+3-MA
TNF-α+EPO
FGF2
22KDa
35KDa
25KDa
15KDa
10KDa
TNF-α
TNF-α+EPO+3-MA
TNF-α+EPO
IGF-1
22KDa
35KDa
25KDa
15KDa
10KDa
TNF-α
TNF-α+EPO+3-MA
TNF-α+EPO

Bax

21KDa

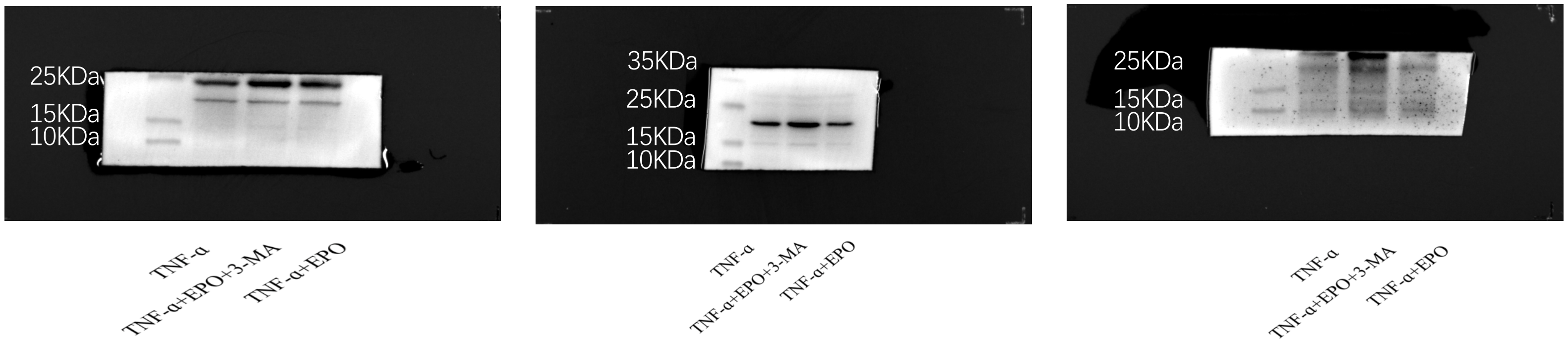

Bcl2

26KDa

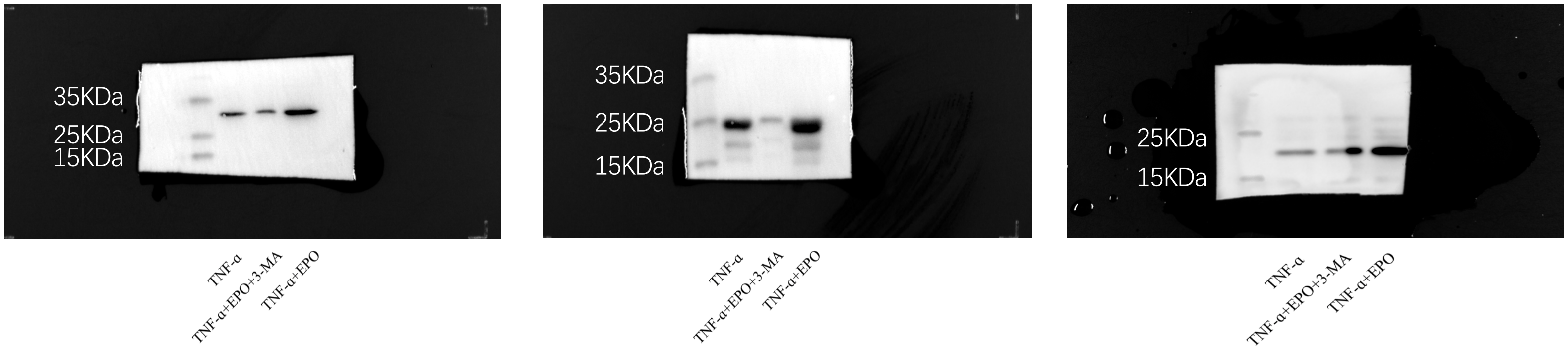

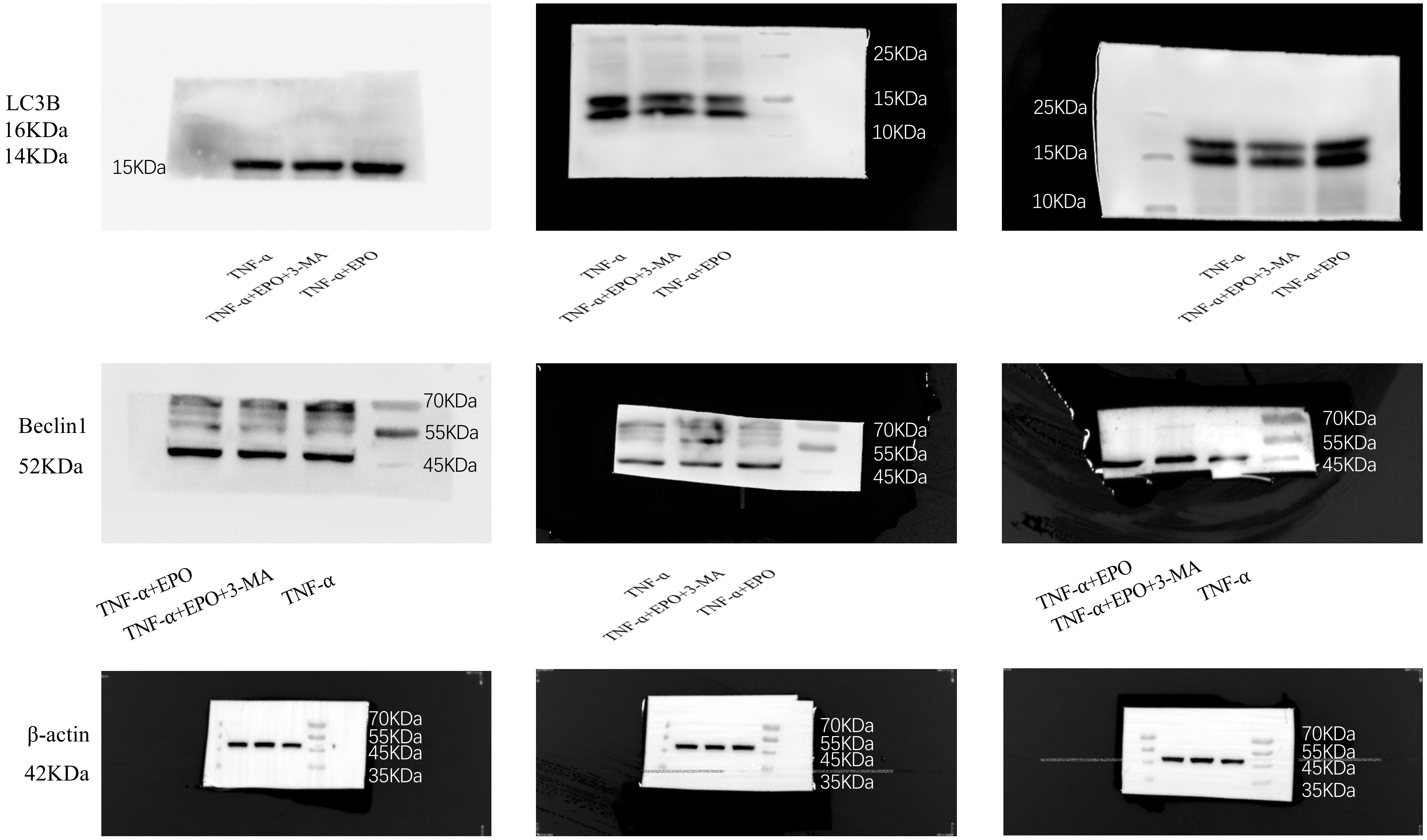
LC3B
16KDa
14KDa
15KDa
25KDa
15KDa
10KDa
25KDa
15KDa
10KDa
TNF-α
TNF-α+EPO+3-MA
TNF-α+EPO
TNF-α
TNF-α+EPO+3-MA
TNF-α+EPO
TNF-α
TNF-α+EPO+3-MA
TNF-α+EPO
Beclin1
52KDa
70KDa
55KDa
45KDa
70KDa
55KDa
45KDa
70KDa
55KDa
45KDa
TNF-α+EPO
TNF-α+EPO+3-MA
TNF-α
TNF-α
TNF-α+EPO+3-MA
TNF-α+EPO
TNF-α+EPO
TNF-α+EPO+3-MA
TNF-α
β-actin
42KDa
70KDa
55KDa
45KDa
35KDa
70KDa
55KDa
45KDa
35KDa
70KDa
55KDa
45KDa
35KDa
