# Supplementary figures and images for: Erythropoietin Activates Autophagy to Regulate Apoptosis and Angiogenesis of Periodontal Ligament Stem Cells via the Akt/ERK1/2/BAD Signaling Pathway under Inflammatory Microenvironment
Source: Stem Cells Int. 2022 Sep 20;2022:9806887. doi: 10.1155/2022/9806887 (PMC9527112; doi:10.1155/2022/9806887)

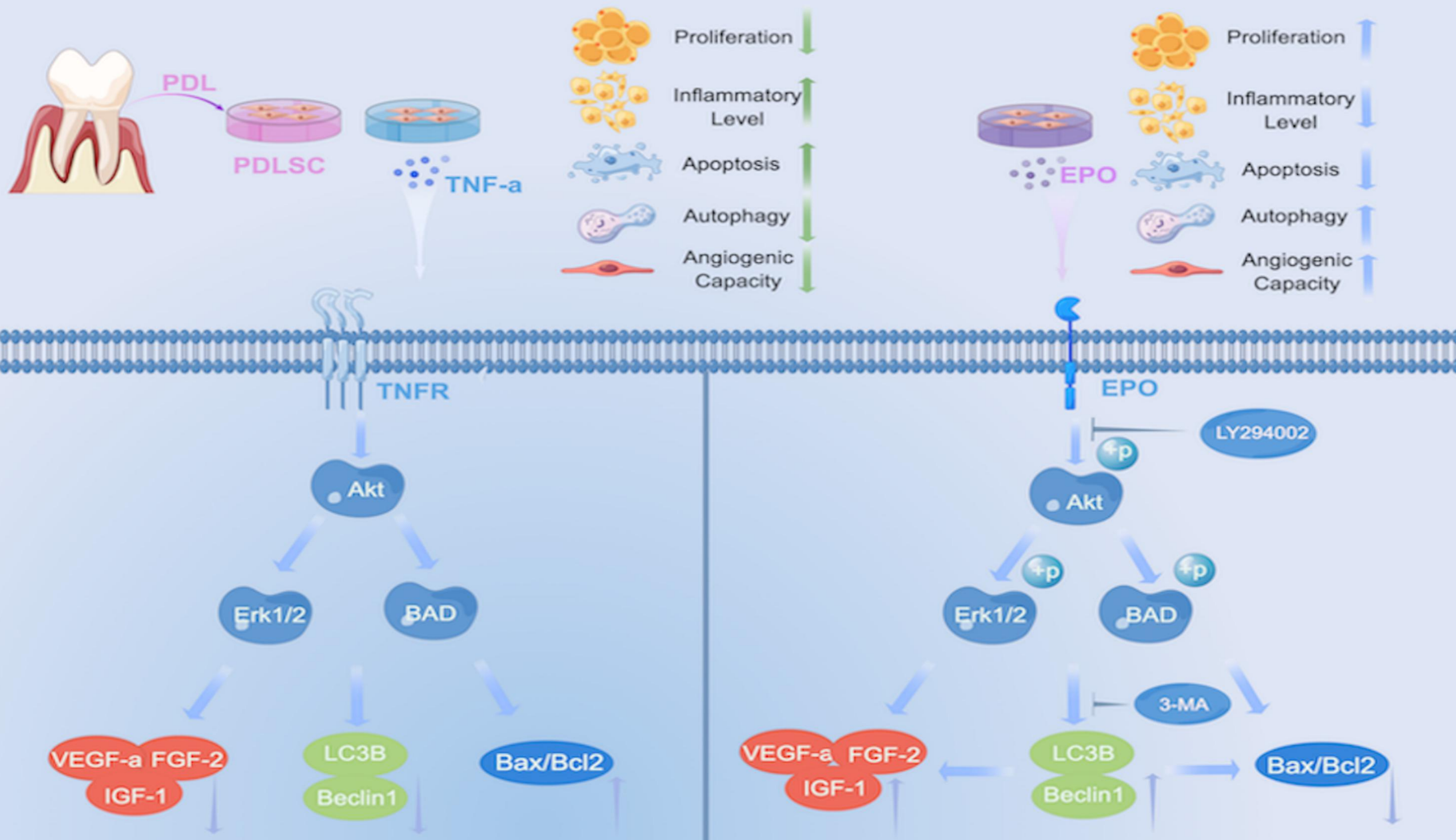

Supplement: Supplementary 2 — Supplementary Figure 2: EPO attenuated inflammation, reduced apoptosis, rescued autophagy, and augmented angiogenesis of PDLSCs under inflammatory microenvironment. EPO activated autophagy to moderate apoptosis and angiogenesis via the Akt/Erk1/2/BAD signaling pathway. [file 9806887.f2.pdf]
